# Targeting neddylation in cancer: molecular mechanisms, translational advances, and therapeutic challenges

**DOI:** 10.3389/fphar.2026.1936781

**Published:** 2026-10-29

**Authors:** Xin Tian, Xinghan Li, Dequan Liu, Ying Min, Yang Hu

**Affiliations:** 1 Department of Stomatology, General Hospital of Northern Theater Command, Shenyang, China; 2 Department of Urology, The First Affiliated Hospital of Dalian Medical University, Dalian, China

**Keywords:** cancer therapy, cullin-RING ligases, Nedd8, neddylation, pevonedistat

## Abstract

Neddylation is a ubiquitin-like post-translational modification that regulates cullin–RING ligases and selected non-cullin substrates involved in cell-cycle progression, DNA replication licensing, DNA-damage repair, apoptosis, hypoxic signaling, inflammatory responses, epithelial–mesenchymal transition, metabolic adaptation, and immune regulation. Many tumors rely on aberrant neddylation-dependent protein turnover to sustain proliferation, survival, metastatic plasticity, and therapeutic resistance, making this pathway a compelling anticancer target. This review outlines the molecular architecture of the NEDD8 cascade, encompassing NEDD8 activation, E2-mediated conjugation, E3/co-E3-facilitated ligation, deneddylation, and the central role of cullin–RING ligases as oncogenic effectors. It also examines the principal classes of neddylation-pathway inhibitors, including the first-in-class NEDD8-activating enzyme inhibitor MLN4924, newer NAE inhibitors such as TAS4464, emerging UBE2M- and UBE2F-directed approaches, DCN1/DCUN1-targeted inhibitors, and natural product-derived or other experimental compounds. Preclinical evidence across hematologic malignancies, gastrointestinal and genitourinary cancers, thoracic and head-and-neck tumors, brain tumors, and pediatric cancers indicates that neddylation inhibition can suppress tumor growth and enhance responses to chemotherapy, radiotherapy, apoptosis-targeted agents, proteasome inhibition, autophagy modulation, and immunotherapy. Nevertheless, clinical translation remains constrained by inadequate patient selection, context-dependent substrate accumulation, adaptive resistance, complex immune effects, and limited therapeutic windows. Future development should therefore move beyond empirical pan-pathway inhibition toward biomarker-guided, tumor-context-specific precision strategies integrating selective E1, E2, and E3 inhibitors, rational combination regimens, pharmacodynamic monitoring, resistance profiling, immune-state assessment, and tumor-targeted delivery systems.

## Graphical Abstract

**Figure:**
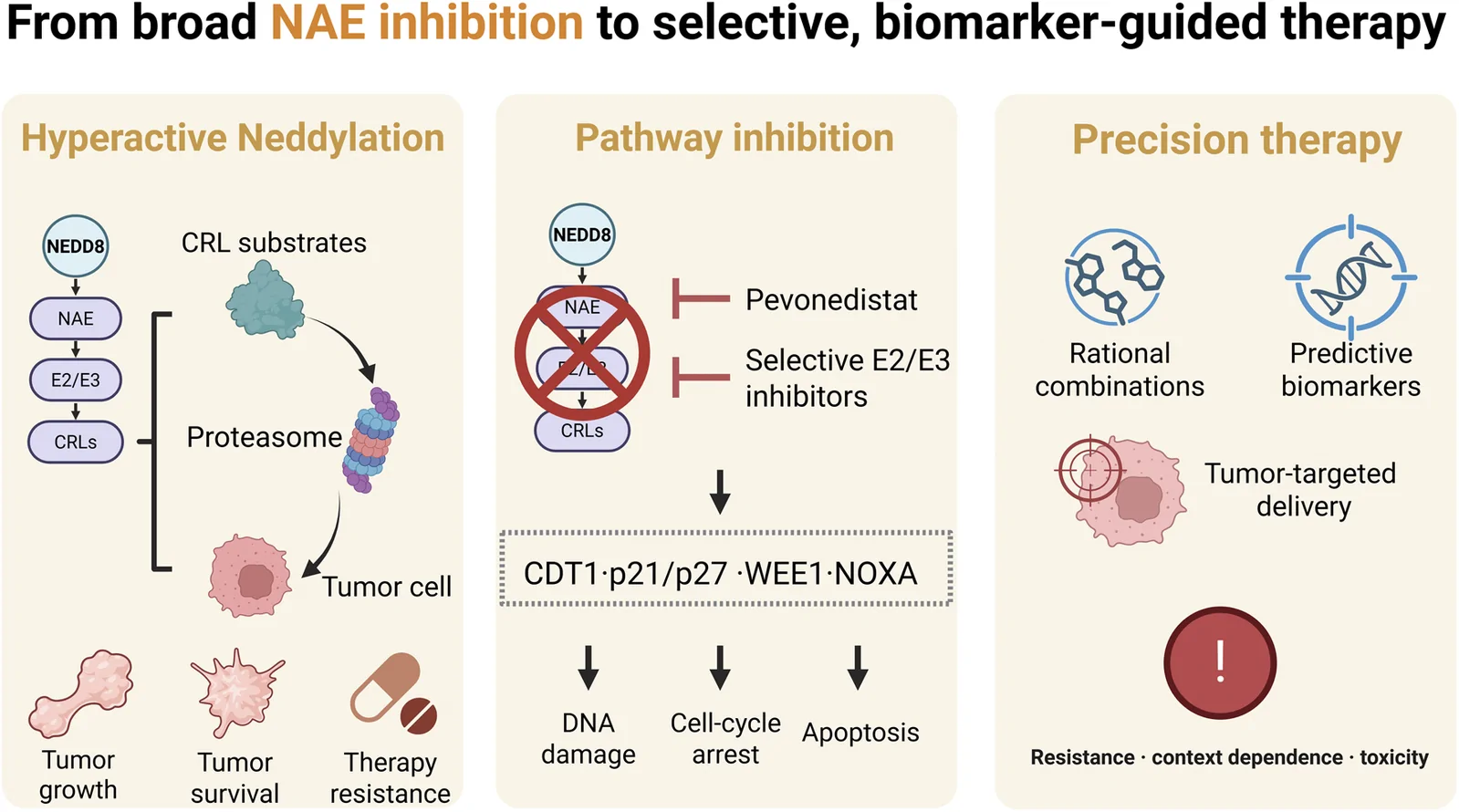
Neddylation-pathway inhibition in precision cancer therapy. Hyperactivated NEDD8–CRL signaling promotes CRL-substrate degradation, thereby supporting tumor growth, survival, and therapeutic resistance. Pevonedistat or selective E2/E3-directed inhibitors suppress this pathway and induce the accumulation of CDT1, p21, p27, WEE1, and NOXA, resulting in DNA damage, cell-cycle arrest, and apoptosis. Future translation should integrate rational combinations, predictive biomarkers, and tumor-targeted delivery to overcome resistance, context-dependent effects, and systemic toxicity. CRL, cullin–RING ligase; NAE, NEDD8-activating enzyme; NEDD8, neural precursor cell expressed, developmentally downregulated 8; NOXA, phorbol-12-myristate-13-acetate-induced protein 1. Created in BioRender. Liu, D. (2026) https://BioRender.com/nwd5xhk.

## 1 Introduction

Neddylation is a ubiquitin-like post-translational modification in which NEDD8 is conjugated to substrate proteins through an E1–E2–E3 enzymatic cascade, thereby regulating cullin-RING ligases (CRLs) and selected non-cullin substrates involved in cell-cycle progression, DNA replication licensing, DNA-damage repair, apoptosis, hypoxia signaling, epithelial–mesenchymal transition, metabolic adaptation, and immune regulation (Zhang S. et al., 2024; Qin X. et al., 2024). Because many tumors depend on hyperactivated CRL-mediated protein turnover to maintain proliferation, survival, metastatic plasticity, and therapy resistance, the neddylation pathway has emerged as a biologically compelling anticancer target (Zhang S. et al., 2024; Qin X. et al., 2024). The first-in-class NEDD8-activating enzyme (NAE) inhibitor MLN4924 (pevonedistat) established that NAE inhibition can suppress cullin neddylation and stabilize CRL substrates, with CDT1 accumulation driving DNA re-replication and subsequent cellular stress (Soucy et al., 2009; Lin et al., 2010). More recent pharmacological progress has extended the field beyond pevonedistat, including orally available NAE inhibitors such as SOMCL-19-133, apoptosis-priming NAE inhibitors such as TAS4464, UBE2F–CRL5 axis inhibitors such as HA-9104, and DCN1/UBE2M interaction inhibitors that provide a conceptual framework for more selective E2-or co-E3-directed neddylation blockade (Zhou LN. et al., 2022; Ochiiwa et al., 2021; Xu T. et al., 2022; Hammill et al., 2018).

Preclinical studies across hematologic malignancies, gastrointestinal cancers, genitourinary tumors, thoracic and head-and-neck cancers, brain tumors, and pediatric cancers indicate that neddylation inhibition can suppress tumor growth, overcome selected drug-resistance programs, and sensitize tumors to chemotherapy, radiotherapy, apoptosis-targeted therapy, or immunotherapy; however, clinical translation remains challenging because pathway inhibition produces highly context-dependent cytotoxic, metabolic, and immune effects (Qin X. et al., 2024; Sekeres et al., 2021). In higher-risk myelodysplastic syndrome (MDS), chronic myelomonocytic leukemia, and low-blast acute myeloid leukemia (AML), pevonedistat plus azacitidine showed encouraging phase II activity but failed to improve event-free survival in the phase III PANTHER trial, emphasizing that broad NAE inhibition alone is insufficient without refined patient selection and pharmacodynamic stratification (Sekeres et al., 2021; Adès et al., 2022). Emerging combination strategies remain promising, as a 2024 phase I study reported that pevonedistat combined with azacitidine and venetoclax was tolerable and showed preliminary activity in relapsed/refractory AML, while mechanistic studies also indicate that MLN4924 can induce PD-L1 through the MEK–JNK–AP1 axis, supporting rational integration with immune-checkpoint or MEK-directed strategies rather than unselected monotherapy (Murthy et al., 2024; Zhang et al., 2022). Overall, we argue that the future of neddylation-targeted cancer therapy should shift from empiric pan-pathway inhibition toward biomarker-guided, tumor-context-specific precision strategies integrating selective E1/E2/E3 inhibitors, rational combinations, pharmacodynamic substrate markers, immune-state assessment, resistance profiling, and tumor-targeted delivery systems (Zhang S. et al., 2024; Qin X. et al., 2024; Murthy et al., 2024; Zhang et al., 2022).

## 2 Central background and rationale of neddylation

Neddylation is a ubiquitin-like post-translational modification in which NEDD8 is covalently attached to substrate proteins through an E1–E2–E3 enzymatic cascade involving the NAE complex NAE1/UBA3, the E2 enzymes UBE2M/UBC12 and UBE2F, and E3 or co-E3 components such as RBX1/RBX2 and DCUN1-family proteins (Zhang S. et al., 2024; Qin X. et al., 2024). Its best-established function is activation of CRLs, which regulate ubiquitin-dependent degradation of numerous substrates involved in cell-cycle progression, DNA replication licensing, DNA damage repair, apoptosis, hypoxia signaling, NF-κB activation, epithelial–mesenchymal transition (EMT), metabolism, and immune regulation (Zhang S. et al., 2024; Qin X. et al., 2024). From a therapeutic perspective, neddylation is attractive because many cancers exhibit enhanced CRL activity and depend on accelerated turnover of tumor-suppressive or stress-regulatory proteins to sustain proliferation, survival, metastatic plasticity, and therapy resistance (Zhang S. et al., 2024; Qin X. et al., 2024). In head and neck squamous cell carcinoma, MLN4924 suppressed migration, invasion, EMT, and tumor growth by disrupting the NEDD8/CUL4/TSC2 axis and inactivating mTOR signaling, supporting a direct link between neddylation and malignant progression (Jiang et al., 2024). In breast cancer models, MLN4924 unexpectedly increased ASCT2/SLC1A5-dependent glutamine uptake through CRL3-SPOP inactivation, and that combined ASCT2 inhibition enhanced antitumor efficacy, indicating that neddylation blockade can expose metabolic adaptive vulnerabilities (Zhou Q. et al., 2022). Additionally, immune effects are also context-dependent. It was demonstrated that MLN4924 induced PD-L1 through the MEK–JNK–AP1 axis and impaired T-cell killing, whereas combination with anti-PD-L1 antibody or MEK inhibition improved antitumor activity, suggesting that neddylation inhibitors may require rational immunotherapy combinations rather than simple monotherapy use (Zhang et al., 2022).

The most clinically advanced strategy is inhibition of NAE by MLN4924, which blocks cullin neddylation, inactivates CRLs (Soucy et al., 2009; Lin et al., 2010). The foundational preclinical rationale was established by Soucy et al., who identified MLN4924 as a selective NAE inhibitor with antitumor activity, and by Lin et al., who showed that CDT1 stabilization drives DNA re-replication, checkpoint activation, apoptosis, and senescence in cancer cells (Soucy et al., 2009; Lin et al., 2010). More recent inhibitor-development studies have expanded the field beyond pevonedistat. It was reported in 2022 that SOMCL-19-133 is a potent, selective, orally available NAE inhibitor that more strongly inhibited cullin neddylation, accumulated CRL substrates, activated Chk1/Chk2, and induced tumor regression in xenograft models compared with MLN4924 (Zhou L. N. et al., 2022). TAS4464 provides another example of mechanistic diversification in 2021 that this NAE inhibitor activated both intrinsic and extrinsic apoptosis in AML through c-Myc-dependent NOXA induction and c-FLIP suppression, leading to complete remission in a human AML xenograft model (Ochiiwa et al., 2021). Clinically, however, the field remains unresolved: a randomized phase II trial suggested that pevonedistat plus azacitidine improved response and event-free survival signals in higher-risk MDS, but the phase III PANTHER trial did not meet its primary event-free survival endpoint in higher-risk MDS/CMML/low-blast AML (Sekeres et al., 2021; Adès et al., 2022). Accordingly, current development should emphasize biomarker selection, tumor-context-specific dependency mapping, rational combinations, and next-generation E1/E2/E3-directed inhibitors.

## 3 Molecular architecture of the neddylation pathway

### 3.1 NEDD8 activation, conjugation, ligation, and removal

Neddylation begins with proteolytic maturation of the NEDD8 precursor to expose the C-terminal diglycine motif required for conjugation. Mature NEDD8 is subsequently activated by the heterodimeric NAE, which comprises NAE1/APPBP1 and UBA3. SENP8/NEDP1 contributes to NEDD8 precursor processing and maintains pathway fidelity by limiting aberrant neddylation of pathway components, including UBE2M/UBC12 (Coleman et al., 2017). Activated NEDD8 is then transferred through a transthiolation reaction to the E2-conjugating enzymes UBE2M/UBC12 or UBE2F, which form partially distinct catalytic modules. UBE2M preferentially cooperates with RBX1 to promote the neddylation of CUL1–4, whereas UBE2F primarily partners with RBX2/SAG to modify CUL5. Supporting the feasibility of selectively targeting individual E2 modules, researchers identified HA-9104 as a selective small-molecule inhibitor of the UBE2F–CRL5 axis (Xu T. et al., 2022; Mamun M. et al., 2023). The final ligation step is coordinated by E3 and co-E3 components, including RBX1/RBX2 and members of the DCUN1 protein family, which facilitate the productive positioning of NEDD8-loaded E2 enzymes toward cullin substrates. Consistent with this co-E3 function, a 2023 study demonstrated that DCUN1D1 promotes prostate cancer cell proliferation and tumor growth by regulating the neddylation of CUL1, CUL3, CUL4A, and CUL5 (Vava et al., 2023). Earlier chemical biology studies further showed that disruption of the DCN1–UBE2M interaction can selectively inhibit DCN1-dependent cullin neddylation (Hammill et al., 2018). Neddylation is dynamically reversible. The COP9 signalosome (CSN), particularly its catalytic CSN5 subunit, functions as the principal deneddylase for cullin substrates and thereby regulates CRL remodeling. SENP8/NEDP1 provides an additional layer of pathway control by removing NEDD8 from aberrantly modified substrates and preventing inappropriate auto- or hyper-neddylation (Coleman et al., 2017; Schulze-Niemand and Naumann, 2023).

### 3.2 Cullin *versus* non-cullin neddylation

Cullins are the predominant and best-characterized substrates of NEDD8. Cullin neddylation functions as a conformational switch that activates CRLs by increasing structural flexibility, facilitating productive E2 engagement, and promoting efficient ubiquitin transfer. Structural studies demonstrated that NEDD8 nucleates a multivalent CRL–UBE2D ubiquitin-ligation complex, whereas structural analyses summarized in 2024 further emphasized that neddylation-dependent conformational dynamics enable functional cooperation between CRLs and additional E3 ligase modules (Baek et al., 2020; Lin and Komives, 2024). Through activation of CRLs, cullin neddylation governs the turnover of numerous substrates involved in cell-cycle progression, DNA replication, apoptosis, Wnt signaling, MAPK/c-Myc signaling, and tumor-cell plasticity. For example, neddylation is required for β-catenin degradation within the Wnt signaling pathway.The UBE2F–CRL5-ASB11–DIRAS2 axis promotes KRAS-driven pancreatic tumorigenesis by targeting the tumor suppressor DIRAS2 for degradation (Wang et al., 2022; Chang et al., 2024). Beyond cancer-cell-intrinsic signaling, cullin neddylation also contributes to immune homeostasis. UBE2M–RBX1 neddylation–CRL module is essential for maintaining regulatory T-cell fitness and suppressive function, underscoring the relevance of neddylation to immune regulation and tumor-microenvironment biology (Wu et al., 2022). Nevertheless, the biological scope of neddylation extends beyond cullin regulation. A growing number of non-cullin proteins, including p53, pVHL, EGFR, E2F1, and MDM2, have been reported as NEDD8 substrates. Depending on the substrate and cellular context, these modifications may influence protein stability, activity, subcellular localization, or protein–protein interactions. Non-cullin neddylation may therefore broaden the functional consequences of the pathway and contribute to the heterogeneous, context-dependent responses observed following pharmacological or genetic inhibition of neddylation (Liu et al., 2024a).

Functionally, these two substrate classes operate at different regulatory levels. Cullin neddylation is an indirect, network-level proteostasis switch: by activating CRLs, it promotes proliferation through the turnover of checkpoint and replication-licensing regulators (Zhang S. et al., 2024; Qin X. et al., 2024), constrains apoptosis through degradation of pro-apoptotic proteins such as NOXA (Zhou W. et al., 2017), and shapes immunity through NF-κB/inflammatory signaling and CRL-dependent maintenance of regulatory T-cell fitness (Wu et al., 2022). Non-cullin neddylation instead acts directly on individual proteins such as p53, MDM2, E2F1, EGFR, and pVHL, altering their stability, transcriptional activity, localization, or molecular interactions. Consequently, its effects on proliferation and apoptosis are substrate- and context-specific, whereas its contribution to immune modulation is less comprehensively defined and appears to arise from discrete signaling nodes rather than wholesale CRL rewiring (Liu et al., 2024a). This distinction also has therapeutic implications: pan-NAE inhibition simultaneously disrupts the dominant cullin-CRL network and selected non-cullin modifications, whereas downstream E2/co-E3 inhibitors may restrict the affected substrate spectrum.

### 3.3 The CRL axis as the core oncogenic effector

The CRL network represents the principal oncogenic output of the neddylation pathway. Within these modular ubiquitin ligase complexes, cullins serve as molecular scaffolds that connect substrate-recognition modules to RBX-family RING proteins and ubiquitin-loaded E2 enzymes, thereby determining substrate selectivity, ubiquitination efficiency, and subsequent proteasomal degradation (Li et al., 2024). Among the CRL family, CUL1-based SKP1–CUL1–F-box (SCF) complexes link neddylation to cell-cycle progression, NF-κB signaling, and adaptation to tumor-associated hypoxia. SCF-SKP2 promotes degradation of the cyclin-dependent kinase inhibitors p27 and p21, thereby facilitating cell-cycle progression. Consistent with this function, a 2024 osteosarcoma study demonstrated that genetic or pharmacological inhibition of SKP2 stabilized p27 and p21, induced apoptosis, suppressed stem-like phenotypes, and prolonged survival in preclinical models (Wang et al., 2024). In colorectal cancer, hypoxia-inducible factor-1α (HIF-1α) binds the CUL1 promoter, promotes formation of an SCF-FBXL1 complex, and facilitates ubiquitin-dependent degradation of the MEN1 tumor suppressor, thereby enhancing tumor-cell proliferation, migration, and tumor growth (Zeng et al., 2022). CUL2 is best characterized as the scaffold of the VHL–Elongin B/C–CUL2–RBX1 complex, which targets hydroxylated HIF-α proteins for proteasomal degradation and thereby couples oxygen sensing to protein turnover. Quantitative reconstitution experiments demonstrated that cullin neddylation accelerates CRL2-VHL-mediated HIF-1α ubiquitination by more than tenfold, providing direct biochemical evidence that neddylation enhances oxygen-dependent HIF regulation (Wang et al., 2021). CUL3 complexes, particularly KEAP1–CUL3–RBX1, regulate NRF2 turnover and cellular redox homeostasis. Mechanistic evidence established KEAP1 as the substrate adaptor responsible for maintaining low basal NRF2 activity through CUL3-dependent degradation; however, the role of CUL3 in cancer appears to be tissue- and subtype-dependent; meanwhile, a breast cancer study identified oncogene-associated features of CUL3 in basal-like and triple-negative breast cancer, whereas tumor-suppressive associations were observed in luminal A disease, highlighting the importance of cellular context in determining the biological consequences of CUL3 activity (Suzuki et al., 2023; Pantazi et al., 2024).

CUL4A- and CUL4B-based ligases broaden the oncogenic output of neddylation to include DNA replication licensing, chromatin regulation, genome maintenance, DNA repair, and therapeutic response. These complexes engage DDB1 and diverse DDB1–CUL4-associated factors (DCAFs) to recognize substrates involved in genomic stability and tumor progression (Cheng et al., 2024). A representative example is CRL4-CDT2-mediated degradation of CDT1, a replication-licensing factor whose timely elimination is required to prevent DNA re-replication. It was demonstrated that SLFN11 interacts with DDB1 and facilitates DDB1–CUL4-CDT2-dependent CDT1 degradation following replicative DNA damage. Loss of SLFN11 impaired this regulatory process and created a synthetic-lethal vulnerability to ATR or CHK1 inhibition (Jo et al., 2021). CUL5 complexes are assembled through Elongin B/C, RBX2/SAG, and SOCS-box-containing substrate receptors. Their oncogenic relevance is exemplified by the UBE2F–SAG–CUL5 axis, in which UBE2F-mediated CUL5 neddylation activates CRL5-dependent degradation of the pro-apoptotic protein NOXA through K11-linked ubiquitination. This mechanism promotes non-small cell lung cancer cell survival and is associated with poor clinical prognosis (Zhou W. et al., 2017). CUL7 differs structurally and mechanistically from canonical CRLs. Cryo-electron microscopy and biochemical analyses published in 2022 showed that CRL7-FBXW8 does not operate as an autonomous neddylated cullin catalytic module. Instead, it functions through an unusual CRL–CRL assembly in which the CUL7–FBXW8 complex is coupled to a neddylated CUL1–RBX1 catalytic module (Hopf et al., 2022). In cancer, CUL7 has been implicated in the context-dependent regulation of IRS-1/PI3K–AKT signaling, p53-associated tumor suppression, epithelial–mesenchymal transition, and cellular stress adaptation. Accordingly, CUL7 may exert either tumor-promoting or tumor-restraining effects depending on tissue origin, substrate-receptor availability, and the surrounding signaling landscape (Shi et al., 2020). Collectively, these findings establish the CRL system as a distributed oncogenic network rather than a single linear signaling cascade.

The molecular structure and drug intervention sites of the NEDD8 cascade mentioned above are shown in Figure 1.

**FIGURE 1:**
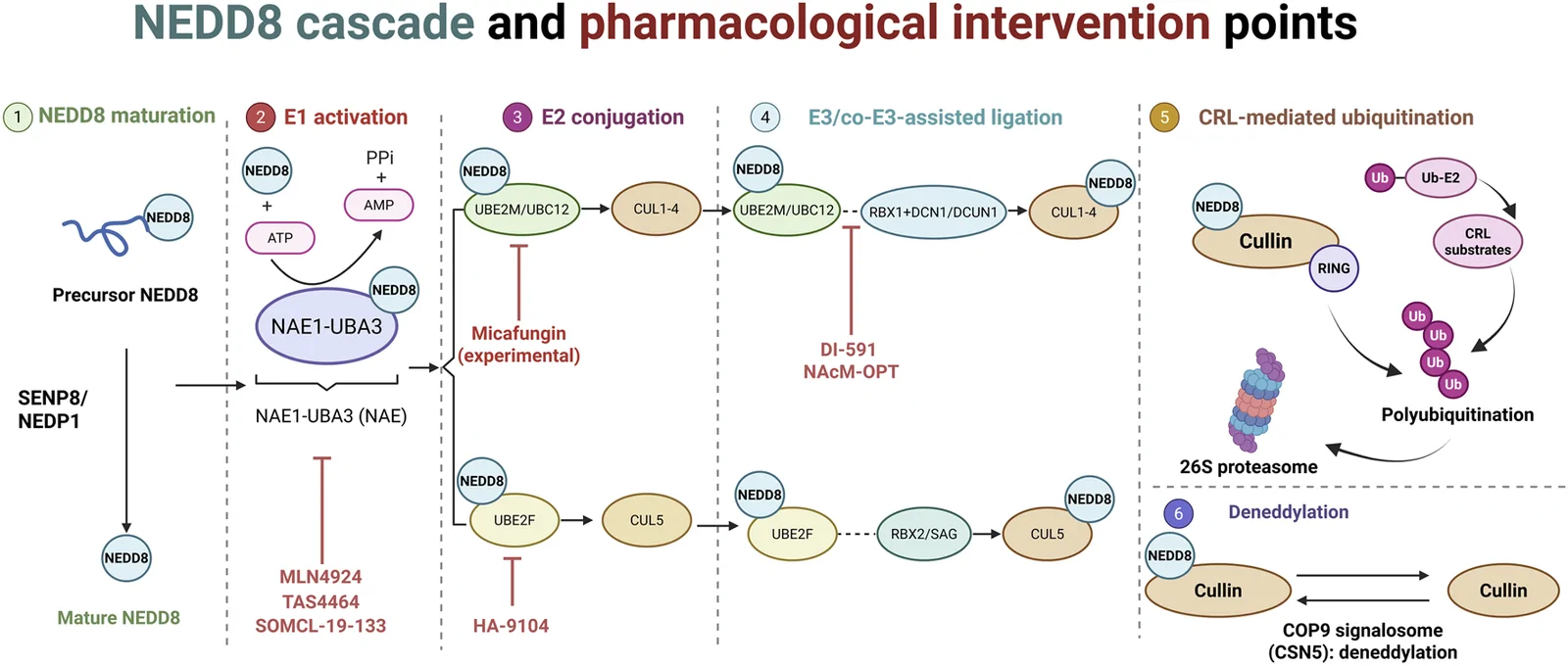
Molecular architecture of the NEDD8 cascade and pharmacological intervention points. NEDD8 precursor maturation by SENP8/NEDP1 exposes the C-terminal diglycine motif required for conjugation. Mature NEDD8 is activated in an ATP-dependent reaction by the heterodimeric NEDD8-activating enzyme NAE1–UBA3 and subsequently transferred to the E2 enzymes UBE2M/UBC12 or UBE2F. UBE2M preferentially cooperates with RBX1 and DCN1/DCUN1-family co-E3 ligases to promote the neddylation of CUL1–4, whereas UBE2F primarily partners with RBX2/SAG to modify CUL5. Cullin neddylation activates cullin–RING ligases, facilitating substrate polyubiquitination and degradation by the 26S proteasome and thereby regulating the cell cycle, DNA replication, apoptosis, hypoxia and redox adaptation, NF-κB signaling, metabolism, and immune responses. The CSN reverses this process through CSN5-mediated deneddylation. Representative pharmacological intervention points include the NAE inhibitors pevonedistat/MLN4924, TAS4464, and SOMCL-19-133; the experimental UBE2M inhibitor micafungin; the UBE2F–CRL5 inhibitor HA-9104; and the DCN1–UBE2M interaction inhibitors DI-591 and NAcM-OPT. Red inhibitory symbols indicate pathway blockade. CRL, cullin–RING ligase; DCN1/DCUN1, defective in cullin neddylation 1; NAE, NEDD8-activating enzyme; NEDD8, neural precursor cell expressed, developmentally downregulated 8; PPi, pyrophosphate; RBX, RING-box protein; SAG, sensitive to apoptosis gene; SENP8/NEDP1, sentrin-specific protease 8/NEDD8-specific protease 1; Ub, ubiquitin; UBE2, ubiquitin-conjugating enzyme E2. Created in BioRender. Liu, D. (2026) https://BioRender.com/d7255lx.

## 4 Classes of neddylation pathway inhibitors

### 4.1 NAE inhibitors

#### 4.1.1 MLN4924

MLN4924, subsequently named pevonedistat, is the first-in-class small-molecule inhibitor of NAE and was developed to pharmacologically suppress NEDD8-dependent CRL activity in cancer cells (Soucy et al., 2009). Unlike a conventional reversible ATP-competitive inhibitor, pevonedistat exploits the catalytic activity of NAE to generate a covalent NEDD8–pevonedistat adduct. This adduct mimics the NEDD8–AMP intermediate, remains tightly bound within the NAE active site, and prevents the productive transfer of activated NEDD8 to the E2 enzymes UBE2M/UBC12 and UBE2F (Brownell et al., 2010; Sarantopoulos et al., 2016). Consequently, pevonedistat rapidly inhibits cullin neddylation and CRL activity, leading to the accumulation of CRL substrates and disruption of multiple cellular processes. Depending on tumor context, these effects can induce DNA-replication stress, cell-cycle arrest, apoptosis, senescence, autophagy, and remodeling of inflammatory signaling pathways (Soucy et al., 2009; Swords et al., 2015). One of the best-characterized consequences of NAE inhibition is stabilization of the replication-licensing factor CDT1. Milhollen et al. demonstrated that impaired CDT1 turnover following NAE inhibition induces DNA re-replication and apoptosis in human tumor cells, thereby establishing a direct mechanistic link between CRL inactivation and lethal replication-licensing stress (Milhollen et al., 2011). Pevonedistat can also activate multiple, context-dependent cell-death and stress-response programs. In liver cancer models, MLN4924 suppresses tumor growth through the concurrent induction of autophagy and apoptosis, suggesting that treatment-induced autophagy may function either as a cytotoxic response or as an adaptive survival mechanism that could be therapeutically cotargeted (Luo et al., 2012a). In pancreatic cancer, a 2022 study further demonstrated that pevonedistat inhibits tumor-cell proliferation and xenograft growth while promoting the accumulation of Wee1, p27, and p21, supporting the concept that tumors with heightened neddylation activity may be vulnerable to enforced checkpoint activation and cell-cycle blockade (Xu J. et al., 2022).

The principal translational advantage of pevonedistat is its capacity to target a pathway-level dependency rather than an individual oncogenic driver. However, its effects vary substantially across tumor types, indicating that effective clinical application will require biomarker-guided patient selection and rational combination strategies. Resistance may arise directly at the drug target. Researchers identified an A171T gatekeeper mutation in UBA3 that reduces MLN4924 binding and is sufficient to confer resistance, demonstrating that genetic alteration of NAE can constitute an on-target resistance mechanism (Toth et al., 2012). Additional studies have expanded the biological consequences of pevonedistat beyond canonical CRL substrate regulation. A 2023 functional-genomic study in retinoblastoma showed that deneddylation of ribosomal proteins contributes to the synergistic interaction between MLN4924 and chemotherapy, suggesting that non-CRL neddylation events may also influence therapeutic response (Aubry et al., 2023). Early-phase clinical studies established the feasibility of administering pevonedistat to patients with AML, MDS, and advanced solid tumors, while demonstrating measurable pharmacodynamic inhibition of the neddylation pathway (Sarantopoulos et al., 2016; Swords et al., 2015). Subsequent clinical development has increasingly focused on combination regimens. A 2024 pediatric phase 1 study evaluated pevonedistat in combination with irinotecan and temozolomide in recurrent or refractory solid and central nervous system tumors, whereas a 2024 phase II trial investigated pevonedistat plus docetaxel in previously treated advanced non-small cell lung cancer (Foster et al., 2024; Qin A. et al., 2024). In 2025, Shoji et al. reported that the combination of pevonedistat, capecitabine, and oxaliplatin was feasible as salvage therapy for advanced gastric cancer. Post-treatment tumor biopsies demonstrated increased CDT1 expression and formation of the pevonedistat–NEDD8 adduct, providing clinical pharmacodynamic evidence consistent with the established mechanism of NAE inhibition (Shoji et al., 2025). Collectively, pevonedistat validated NAE and the broader neddylation pathway as pharmacologically tractable targets in cancer. Nevertheless, its clinical development has also revealed the limitations of indiscriminate pathway inhibition. Future progress will depend on identifying neddylation-dependent tumor states.

#### 4.1.2 TAS4464

TAS4464 is a potent and selective NAE inhibitor developed after MLN4924 with the aim of achieving more sustained suppression of neddylation signaling. By inhibiting NAE-dependent NEDD8 activation, TAS4464 prevents cullin neddylation, disrupts CRL activity, and impairs CRL-mediated substrate turnover (Yoshimura et al., 2019). In the foundational preclinical study, TAS4464 inhibited NAE more potently than MLN4924 in both biochemical and cellular assays. Treatment markedly suppressed cullin neddylation and promoted the accumulation of canonical CRL substrates, including CDT1, p27, and phosphorylated IκBα, thereby providing a pharmacodynamic basis for its antiproliferative activity (Yoshimura et al., 2019). TAS4464 also exhibited broad cytotoxicity across diverse human cancer cell lines and patient-derived tumor cells. Notably, prolonged target inhibition and marked antitumor activity were observed in hematologic and solid-tumor xenograft models after once- or twice-weekly administration, indicating that sustained pathway suppression may contribute to its preclinical efficacy (Yoshimura et al., 2019). Subsequent studies identified tumor-lineage-specific mechanisms that extend beyond generalized CRL inactivation. In multiple myeloma, TAS4464 suppresses both canonical and noncanonical NF-κB signaling, thereby disrupting a survival pathway that is central to plasma-cell malignancy biology (Muraoka et al., 2019). In AML, TAS4464 induces apoptosis through a c-Myc-dependent signaling program characterized by NOXA upregulation, c-FLIP suppression, and activation of both the caspase-9-mediated intrinsic pathway and the caspase-8-mediated extrinsic pathway (Ochiiwa et al., 2021). The same study demonstrated complete tumor remission in a human AML xenograft model, suggesting that, in selected molecular contexts, CRL inhibition can be translated into coordinated activation of multiple apoptotic pathways (Ochiiwa et al., 2021). These findings position TAS4464 as a mechanistically informative NAE inhibitor whose efficacy may depend on lineage-specific vulnerabilities, including NF-κB dependence in multiple myeloma and c-Myc-associated apoptotic priming in AML.

Despite its strong preclinical activity, clinical evaluation revealed a substantial discrepancy between pharmacological potency and therapeutic tolerability. TAS4464 entered first-in-human testing in an open-label phase I study involving patients with advanced or metastatic solid tumors (Yamamoto et al., 2021). Dose escalation began with a once-weekly regimen at 10 mg/m^2^, and a 14-day lead-in schedule was subsequently introduced in response to treatment-associated abnormalities in liver-function tests. Nevertheless, hepatic laboratory abnormalities and gastrointestinal toxicities remained the most frequent treatment-related adverse events (Yamamoto et al., 2021). Dose-limiting toxicities occurred at 56 mg/m^2^, and the maximum tolerated dose could not be established. Further escalation was discontinued because serious treatment-related adverse events, treatment discontinuations, and dose-limiting toxicities were predominantly associated with hepatic dysfunction (Yamamoto et al., 2021). Clinical antitumor activity was also limited. No complete or partial responses were observed, and only one patient achieved prolonged stable disease, indicating that potent systemic NAE inhibition did not translate into broad single-agent efficacy in an unselected solid-tumor population (Yamamoto et al., 2021). Accordingly, researchers have characterized TAS4464 as an important proof-of-concept compound that validates the biological consequences of sustained NAE inhibition while simultaneously exposing unresolved challenges related to therapeutic window, hepatotoxicity, dose scheduling, patient selection, and pharmacodynamic monitoring (Qin X. et al., 2024; Fu and Wang, 2023). The future significance of TAS4464 may therefore lie primarily in its contribution to next-generation drug development. Its preclinical and clinical profiles support the pursuit of NAE inhibitors with improved safety margins, as well as more selective approaches targeting individual E2, E3, or CRL modules.

### 4.2 E2 enzyme inhibitors: UBE2M and UBE2F as emerging targets

Compared with global inhibition of NAE, direct or interface-directed targeting of the NEDD8 E2 enzymes UBE2M/UBC12 and UBE2F may provide greater pathway selectivity because these enzymes channel neddylation into partially distinct cullin modules rather than suppressing the entire NEDD8 cascade (Stanley et al., 2012; Zhou et al., 2023). UBE2M preferentially cooperates with RBX1 and DCUN1-family co-E3 proteins to promote the neddylation of CUL1, CUL2, CUL3, CUL4A, and CUL4B, whereas UBE2F primarily partners with RBX2/SAG to regulate CUL5 neddylation (Stanley et al., 2012; Zhou et al., 2023). This biochemical divergence provides a rationale for selectively targeting CRL1–4- or CRL5-dependent tumor states. UBE2M-centered signaling influences cell-cycle progression, DNA damage responses, senescence, apoptosis, NRF2 regulation, and immune homeostasis, whereas UBE2F-dependent signaling is more closely associated with CUL5-mediated suppression of apoptosis and resistance to chemotherapy and radiotherapy (Wu et al., 2022; Zhou et al., 2023). Recent studies have identified context-specific oncogenic functions of UBE2M. In estrogen receptor-positive breast cancer, UBE2M participates in a positive feedback circuit with estrogen receptor-α (ERα). ERα enhances HIF-1α-dependent transcription of UBE2M, whereas UBE2M promotes ERα stability through a UBE2M–CUL3/4A–E6AP–ERα regulatory axis, thereby facilitating tumor progression and resistance to fulvestrant (Lin et al., 2024). In colorectal cancer, UBE2M promotes G2/M progression by neddylating USP39, which regulates PABPC1 deubiquitination, enhances CCNB1 translation, and accelerates cell-cycle progression. Notably, the same study identified micafungin as a candidate UBE2M-targeting inhibitor capable of disrupting this signaling axis (Wang Z. et al., 2026). Consistent with this pharmacological direction, micafungin directly interacts with UBE2M, suppresses neddylation, promotes CRL-substrate accumulation, reduces cancer-cell survival and migration, and induces DNA damage. However, its translational potential as an anticancer UBE2M inhibitor remains to be established (Mamun MAA. et al., 2023).

Current UBE2M-directed drug discovery has focused primarily on disrupting the interaction between UBE2M and the co-E3 protein DCN1 rather than directly targeting the catalytic cysteine of UBE2M. This strategy exploits the defined hydrophobic pocket through which DCN1 recognizes the acetylated N terminus of UBE2M (Scott et al., 2017). Scott et al. demonstrated in 2017 that blocking this N-terminal-acetylation-dependent DCN1–UBE2M interaction selectively impairs DCN1-dependent cullin neddylation, while Hammill et al. developed orally bioavailable DCN1 inhibitors that disrupt DCN1–UBE2M binding and suppress DCN1-mediated cullin neddylation in biochemical and cellular systems (Hammill et al., 2018; Scott et al., 2017). Further optimization generated pyrazolo-pyridone DCN1 inhibitors with improved chemical properties and selective activity against CUL1 and CUL3 neddylation in DCN1-amplified squamous carcinoma models. These findings support the concept that targeting E2/co-E3 interfaces may achieve partial CRL selectivity while avoiding complete pathway shutdown (Kim et al., 2019). Although UBE2F has been less extensively investigated than UBE2M, it represents an attractive target in tumors that depend on suppression of mitochondrial apoptosis. UBE2F activates the CRL5 complex and promotes K11-linked ubiquitination and degradation of the pro-apoptotic protein NOXA, thereby enhancing lung cancer cell survival (Zhou W. et al., 2017). Follow-up studies showed that platinum exposure and ionizing radiation increase the functional dependence of lung cancer cells on UBE2F–CUL5 signaling. Accordingly, genetic depletion of UBE2F restored NOXA-dependent apoptosis and increased sensitivity to platinum-based chemotherapy and radiotherapy (Zhou L. et al., 2020; Zhou L. et al., 2021). Direct pharmacological validation was provided by Xu et al., in 2022 through the identification of HA-9104, a small-molecule inhibitor of the UBE2F–CRL5 axis. HA-9104 suppressed CUL5 neddylation and induced DNA damage and G2/M arrest; proposed DNA-interacting activity of its 7-azaindole moiety may also contribute to the latter effects, although direct DNA binding was not demonstrated (Xu T. et al., 2022). Collectively, UBE2M and UBE2F represent promising E2-level targets for achieving greater selectivity within the neddylation pathway. UBE2M-directed approaches may be particularly relevant to tumors driven by CRL1–4-dependent cell-cycle, transcriptional, or stress-response programs, whereas UBE2F inhibition may preferentially restore apoptotic sensitivity in CUL5-dependent tumors. Nevertheless, the field remains at a predominantly preclinical stage, and no UBE2M- or UBE2F-targeted inhibitor has yet achieved the clinical maturity of NAE inhibitors.

### 4.3 E3/co-E3 inhibitors: DCUN1/DCN1-targeted strategies

DCUN1D1, also known as DCN1 or SCCRO, functions as a scaffold-type NEDD8 co-E3 ligase that enhances cullin neddylation by orchestrating three core components: cullin scaffolds, RBX1-containing CRL complexes, and NEDD8-loaded UBE2M/UBC12 (Kurz et al., 2008; Kim et al., 2008). At the molecular level, DCN1 recognizes the N-terminally acetylated methionine of UBE2M through a conserved hydrophobic pocket, thereby promoting the productive orientation of the NEDD8 transfer machinery toward cullin substrates (Kim et al., 2008; Scott et al., 2011). Mammalian cells express multiple DCN-like proteins with overlapping but distinct patterns of subcellular localization and cullin preference, indicating that DCUN-family co-E3 ligases provide an additional level of spatial and substrate selectivity within the neddylation cascade (Keuss et al., 2016). This feature distinguishes DCN1-directed inhibition from pan-NAE blockade. Rather than suppressing the entire NEDD8 pathway, inhibitors of the DCN1–UBE2M interface are designed to disrupt a defined protein–protein interaction and selectively attenuate specific cullin neddylation events (Zhang S. et al., 2024; He et al., 2024). The oncologic rationale for targeting DCN1 is supported by recurrent amplification or overexpression of DCUN1D1 in several tumor types, particularly squamous malignancies. In non-small cell lung cancer, DCUN1D1 enhances metastatic progression through activation of focal adhesion kinase signaling and upregulation of PD-L1, linking DCN1 activity to invasive behavior and suggesting a possible connection to immune evasion (Li J. et al., 2019). These findings suggest that DCN1 inhibition may be particularly relevant in tumors characterized by DCUN1D1 amplification, CUL3/KEAP1–NRF2 dependence, aberrant Wnt/β-catenin signaling, or immune-evasive phenotypes sustained by selective cullin neddylation (Zhang S. et al., 2024; Vava et al., 2023; Li J. et al., 2019).

Small-molecule inhibitors of the DCN1–UBE2M/UBC12 interaction provide the strongest pharmacological support for this co-E3-directed strategy. DI-591, developed through structure-guided optimization, is a high-affinity, cell-permeable inhibitor that binds DCN1/DCN2 and disrupts their interaction with UBC12 in cells (Zhou H. et al., 2017). DI-591 preferentially converted CUL3 to an unneddylated, inactive state while exerting minimal effects on other cullins, thereby establishing that pharmacological inhibition of DCN1 can achieve cullin-selective suppression rather than reproduce the broad consequences of NAE inhibition (Zhou H. et al., 2017). Independent chemical-biology studies targeting the same N-acetyl-UBE2M recognition pocket further validated the DCN1–UBE2M interface as a druggable protein–protein interaction (Scott et al., 2017). Subsequent medicinal-chemistry efforts improved the pharmacological properties and mechanistic diversity of DCN1 inhibitors. NAcM-OPT, an optimized piperidinyl-urea derivative, retained potent biochemical and cellular activity while exhibiting improved metabolic stability and oral bioavailability, establishing it as a useful *in vivo* probe for acute inhibition of DCN1–UBE2M signaling (Hammill et al., 2018). Pyrazolo-pyridone inhibitors reported in 2019 selectively reduced steady-state CUL1 and CUL3 neddylation in DCN1-amplified squamous carcinoma models, further supporting the potential application of partial CRL inhibition in molecularly selected tumors (Kim et al., 2019). In 2021, covalent DCN1 inhibitors targeting a cysteine residue within the ligand-binding pocket were also shown to preferentially suppress CUL3 neddylation, demonstrating the chemical feasibility of irreversible, CUL3-biased DCN1 inhibition (Zhou H. et al., 2021). Despite these advances, DCN1-targeted therapy remains at the preclinical stage.

### 4.4 Natural products and experimental neddylation inhibitors

Natural products and screening-derived compounds share several preclinical properties: they partially suppress NEDD8 conjugation or cullin activation, perturb SKP2-centered CRL signaling, and inhibit tumor growth in experimental systems. However, most have micromolar potency, pleiotropic actions, and incompletely resolved direct targets and should therefore be regarded as chemical probes or lead compounds rather than clinically validated neddylation-targeted therapeutics. Their mechanistic distinctions are more informative than the shared downstream cell-cycle and death phenotypes. Gossypol directly binds SAG-CUL5 and RBX1-CUL1 complexes, suppressing CUL5 and CUL1 neddylation and altering the corresponding CRL substrates NOXA and MCL-1 (Yu et al., 2020). Flavokawain A is distinguished by UBC12/CUL1 deneddylation and by validation in the TRAMP prostate-cancer model and prostate tumor-initiating-cell systems (Li et al., 2015; Song et al., 2022). Flavokawain B interacts with the APPBP1/NAE1 regulatory subunit and has been evaluated principally as a bortezomib sensitizer in prostate-cancer models (Li X. et al., 2019). Gartanin has no conclusively established direct NEDD8-cascade target; its distinguishing features are FBXW2 upregulation and autophagy in prostate-cancer models (Pham et al., 2020). Natural-product-like discovery has additionally yielded 6,6″-biapigenin, a micromolar NAE inhibitor identified by virtual screening, and a dipeptide-conjugated deoxyvasicinone derivative that inhibits NAE with selectivity over related ubiquitin-like E1 enzymes, with molecular modelling suggesting competition at the ATP-binding site (Leung et al., 2011; Zhong et al., 2012).

Beyond natural products, several repurposed or experimentally derived compounds have been identified through biochemical screening, virtual screening, structure-guided design, and cell-based approaches. Piperacillin was reported in 2014 as an FDA-approved drug with NAE-inhibitory activity identified through structure-based screening, thereby demonstrating the feasibility of using approved-drug libraries to discover NEDD8-pathway modulators. This report provides preclinical evidence for repurposing; it does not establish clinical anticancer efficacy (Zhong et al., 2014). Mitoxantrone was similarly repurposed in 2018 as an ATP-competitive NAE inhibitor that suppresses NAE activity in biochemical and cellular assays and induces apoptosis through stabilization of the neddylation-regulated substrate p53. Nevertheless, its established pleiotropic cytotoxic activity complicates attribution of its antitumor effects specifically to NAE inhibition (Wu et al., 2018). Candesartan cilexetil was identified in 2020 through screening of 1,331 approved drugs as an ATP-competitive NAE inhibitor. The compound suppressed cullin neddylation, induced apoptosis, and inhibited A549 lung cancer xenograft growth, supporting its potential as a repurposing-derived lead rather than an immediately applicable neddylation-targeted therapy (Ni et al., 2020). HA-1141, identified in 2021 through virtual screening and structural optimization, directly binds NAE and inhibits the neddylation of CUL1–5. However, treatment also induces noncanonical endoplasmic reticulum stress, activates the PKR-mediated integrated stress response, suppresses mTORC1 signaling, and promotes autophagy, illustrating the pleiotropic biological effects that may accompany experimental neddylation inhibition (Li et al., 2021). Collectively, natural-product-derived, repurposed, and screening-generated inhibitors have substantially expanded the chemical and mechanistic landscape of neddylation-targeted drug discovery. Their principal value currently lies in pathway interrogation, identification of unconventional inhibitory mechanisms, and generation of lead structures for medicinal-chemistry optimization. Clinical relevance remains preliminary because many of these compounds exhibit only micromolar potency, incomplete target-selectivity profiles, uncertain pharmacokinetic behavior, limited toxicity characterization, and insufficient validation in disease-relevant *in vivo* models or human trials.

### 4.5 Shared cellular consequences of neddylation-pathway inhibition

Across tumor lineages, inhibition of NAE or downstream E2/co-E3 modules converges on a common set of cellular consequences that should be distinguished from tumor-specific dependencies. CRL inactivation stabilizes replication-licensing and checkpoint substrates, including CDT1, p21, p27, and WEE1, thereby producing DNA re-replication or replication stress, checkpoint activation, and cell-cycle arrest (Soucy et al., 2009; Lin et al., 2010). NOXA accumulation can favor apoptosis in UBE2F–CUL5-dependent lung cancer models (Zhou W. et al., 2017), Suppression of NF-κB signaling contributes to MLN4924 activity in activated B-cell-like DLBCL (Milhollen et al., 2010). Sustained stress may induce senescence or autophagy; protective autophagy was demonstrated in liver cancer models (Luo et al., 2012a). Other recurring effects include impaired DNA repair, reduced epithelial-mesenchymal transition and invasion, altered redox or metabolic adaptation, and context-dependent remodeling of cytokine production, antigen presentation, and immune-cell function (Liu et al., 2024a; Zhou L. et al., 2019). These shared responses explain the broad antitumor activity of neddylation inhibitors but do not, by themselves, define a tumor-selective vulnerability. Accordingly, Section 5 emphasizes lineage-specific axes, biomarkers, and combination opportunities rather than repeating this common p21/p27/CDT1-associated stress-and-death program.

## 5 Preclinical evidence across cancer types

### 5.1 Hematologic malignancies

Among hematologic malignancies, AML illustrates a lineage-defined neddylation dependency rather than a generic consequence of CRL shutdown. Early work showed that MLN4924 overcomes stromal survival signaling in AML (Swords et al., 2010). In FLT3-ITD AML, the more distinctive mechanism is suppression of the NF-κB-miR-155 program, restoration of SHIP1 and PU.1, and induction of myeloid differentiation; in parallel, NOXA induction creates a BCL-2-family vulnerability that supports combination with apoptosis-targeted agents (Khalife et al., 2015; Knorr et al., 2015). Azacitidine further augments NOXA, providing a mechanistic basis for combining pevonedistat with azacitidine and venetoclax (Cojocari et al., 2022). The biological continuity between high-risk MDS and AML, together with the ability to assess pathway inhibition in blood and marrow, supported early clinical translation (Swords et al., 2015). Thus, the tumor-specific rationale in AML/MDS centers on FLT3-ITD/NF-κB-miR-155 differentiation control and NOXA-dependent apoptotic priming.

Across lymphoid malignancies, response is determined by lineage-specific survival circuitry. In activated B-cell-like DLBCL, NAE inhibition principally suppresses NF-κB signaling, whereas germinal-center B-cell-like DLBCL exhibits CDT1-linked replication stress, providing a mechanistically discriminative subtype comparison (Milhollen et al., 2010). In mantle-cell lymphoma, neddylation blockade lowers BCL-xL and sensitizes tumors to rituximab (Czuczman et al., 2016). MLN4924 induces cell-line-dependent apoptosis or senescence, accompanied by changes in BIK/NOXA and IAP-family proteins or accumulation of p21/p27, respectively (Wang et al., 2015). Multiple myeloma is distinguished by dependence on canonical and noncanonical NF-κB signaling and proteostasis, explaining the activity of TAS4464 with bortezomib, immunomodulatory therapy, and antibody-based regimens (Muraoka et al., 2019). In CLL, the relevant vulnerability is microenvironment-driven NF-κB signaling and BIM suppression (Godbersen et al., 2014). Limited single-agent activity in early clinical evaluation reinforces the need for lineage and molecular selection (Shah et al., 2016).

### 5.2 Gastrointestinal cancers

Gastrointestinal cancers are better differentiated by tumor-specific axes. In colorectal cancer, concurrent TOP1 and NEDD8 inhibition increases the persistence and toxicity of TOP1-DNA lesions in cell lines, patient-derived organoids, and xenografts (Mi et al., 2025; Sun et al., 2023), whereas the UBE2M-USP39-PABPC1-CCNB1 axis promotes G2/M progression through post-transcriptional control of cyclin B1 (Wang Z. et al., 2026). In colorectal cancer cell models, p53 proficiency enhanced MLN4924-induced apoptosis, while depletion of the anti-apoptotic protein FLIP further sensitized cells to MLN4924 (Ferris et al., 2020). In gastric cancer, a distinctive mechanism links microbial infection to ferroptotic sensitivity. *Helicobacter pylori* downregulates NAE1, thereby reducing NAE1-mediated neddylation and stabilization of transferrin receptor 1 and limiting erastin-induced ferroptosis; restoration of NAE1 increases ferroptotic sensitivity and suppresses metastatic phenotypes (Wang et al., 2025). This axis extends earlier evidence that gastric cancer is neddylation-dependent (Lan et al., 2016). In KRAS-mutant pancreatic cancer, UBE2F-CRL5-ASB11 drives degradation of the tumor suppressor DIRAS2 (Chang et al., 2024), while gemcitabine and radiation combinations exploit therapy-specific checkpoint and damage-response interactions (Li et al., 2017; Wei et al., 2012).

In hepatocellular carcinoma, UBE2M-centered signaling is linked to β-catenin/cyclin D1 and p53/MDM2 regulation, nominating E2-level intervention as a disease-relevant alternative to global NAE inhibition (Zhang et al., 2020; Kim et al., 2021). In intrahepatic cholangiocarcinoma, NEDD8, NAE1, UBA3, and UBC12 are frequently upregulated; pathway blockade suppresses tumor growth and supports combination with cisplatin (Gao et al., 2014). A complementary study identified NAE1-dependent hyperneddylation as a regulator of tumor-stroma crosstalk (Olaizola et al., 2022). These axes, rather than the shared checkpoint-substrate response, define the most informative gastrointestinal vulnerabilities.

### 5.3 Genitourinary tumors

Genitourinary tumors reveal distinct metabolic, hormonal, and therapy-response dependencies. In ccRCC/KIRC, earlier studies established pathway activation and associated neddylation-related programs with inflammatory and immune states (Tong et al., 2017; Liu et al., 2024b). The most disease-specific mechanism is the CUL4B-FBP1 axis. CUL4B-dependent degradation of nuclear FBP1 sustains HIF-regulated GLUT1, LDHA, PDK1, and VEGF expression; MLN4924 restores nuclear FBP1, reduces glucose uptake, lactate production, and NADPH generation, and sensitizes VHL-deficient ccRCC tumors to buthionine sulfoximine *in vivo* (Yang Y. et al., 2025). This axis links neddylation to the defining VHL-HIF metabolic biology of ccRCC rather than only to generic checkpoint-substrate accumulation.

In bladder cancer, the most actionable axis is chemotherapy sensitization: MLN4924 enhances cisplatin-induced damage through JNK activation, Bcl-xL downregulation, and caspase engagement (Kuo et al., 2015; Ho et al., 2015), while a neddylation-gene signature has been proposed for outcome stratification but requires prospective validation (Xiaolong et al., 2025). Prostate cancer is distinguished by repression of AR/AR-V7 transcription and invasion, supporting combinations with androgen-axis therapy (Wang et al., 2014; Zhou X. et al., 2020), and by a UBC12-WEE1 checkpoint dependency that can be exploited during radiotherapy (Wang et al., 2016). In ovarian cancer, neddylation blockade can restore platinum sensitivity (Jazaeri et al., 2013; Nawrocki et al., 2013), yet it may antagonize paclitaxel-induced microtubule polymerization (Hong et al., 2019). This contrast makes treatment-partner mechanism, rather than generic cytotoxicity, the central determinant of combination design.

### 5.4 Thoracic and head-and-neck cancers

Thoracic malignancies are best distinguished by E2-CRL dependencies. In NSCLC, UBE2F partners with RBX2/SAG to neddylate CUL5, enabling K11-linked ubiquitination and degradation of NOXA (Zhou W. et al., 2017). Platinum exposure and ionizing radiation reinforce reliance on this branch; UBE2F depletion restores NOXA-dependent apoptosis and sensitizes lung-cancer cells to both therapies (Zhou L. et al., 2020; Zhou L. et al., 2021), while HA-9104 pharmacologically validates the UBE2F-CRL5 axis and enhances radiosensitivity (Xu T. et al., 2022). A separate UBE2M vulnerability is supported by its overexpression in lung cancer and by the activity of UBE2M depletion in MLN4924-resistant cells (Li L. et al., 2019). In SCLC and extrapulmonary small-cell carcinoma, the relevant dependency is maintenance of the neuroendocrine lineage state (Norton et al., 2021). Thus, the lung-cancer rationale centers on the UBE2F-CUL5-NOXA axis, UBE2M-mediated resistance, and lineage identity.

In HNSCC, tumor-specific combination mechanisms include replication-associated radiosensitization (Vanderdys et al., 2018), a c-Myc-NOXA death program (Zhang et al., 2019), and stabilization of TOP1 that increases sensitivity to TOP1-directed therapy (Gu et al., 2023). DCUN1D1/DCN1 provides a biomarker-defined opportunity across thoracic and head-and-neck squamous cancers because it lies within the recurrent 3q26 amplicon (Sarkaria et al., 2006), is frequently amplified in LUSC (Chatziandreou et al., 2021), and can be engaged by DCN1-UBE2M interaction inhibitors that preferentially reduce CUL1/CUL3 neddylation in DCN1-amplified squamous models (Kim et al., 2019). These features support selective, biomarker-enriched DCN1 targeting rather than undifferentiated NAE inhibition.

### 5.5 Brain tumors and pediatric tumors

Glioblastoma provides the strongest brain-tumor rationale for neddylation targeting, with pathway hyperactivation documented in experimental disease models (Hua et al., 2015). Patient-derived stem-like cells with ERK/AKT activation appear particularly sensitive (Han et al., 2019), whereas treatment-induced PD-L1 creates an adaptive immune-checkpoint dependency (Zhou S. et al., 2019). Additional tumor-specific opportunities include re-sensitization of temozolomide-resistant cells and increased susceptibility to oncolytic vesicular stomatitis virus (Brandt et al., 2023; Guo, 2023). These data position glioblastoma as a combination-focused indication defined by oncogenic signaling state and adaptive immune or viral vulnerabilities.

Pediatric tumors also require lineage-specific interpretation. In neuroblastoma, p53 status modulates the cellular response to pevonedistat without being an absolute determinant of orthotopic xenograft efficacy (Foster et al., 2021), and Pediatric Preclinical Testing Program data demonstrate marked heterogeneity across childhood tumor models (Smith et al., 2012). In group 3/4 medulloblastoma, mutant KBTBD4 creates a neomorphic CRL3 adaptor that drives CoREST degradation, nominating adaptor-selective biology as a more precise vulnerability (Chen et al., 2022). The pediatric phase I combination of pevonedistat, irinotecan, and temozolomide established a recommended phase II dose but did not establish comparative efficacy (Foster et al., 2024). In retinoblastoma, intravitreal delivery exploits a localized SKP2-CRL dependency while limiting systemic exposure (Aubry et al., 2020). Collectively, pediatric development should be guided by developmental lineage, CRL-adaptor specificity, molecular subtype, and feasible delivery route. Table 1 summarizes the key neddylation-related vulnerabilities, anti-tumor mechanisms, rational combination therapy strategies, and current progress in translational research regarding various representative hematological malignancies and solid tumors discussed above.

**TABLE 1:** Tumor-specific biological functions, therapeutic mechanisms, and translational prospects of neddylation-pathway inhibition.

Tumor category	Representative malignancies	Major neddylation-related targets or dependencies	Principal biological and antitumor effects of pathway inhibition	Promising therapeutic strategies	Current evidence and translational status	Representative references
Hematologic malignancies	AML and high-risk MDS	NAE–CRL signaling; NF-κB; miR-155–SHIP1/PU.1 axis; CDT1; NOXA; BCL-2-family dependence	Suppresses the FLT3-ITD/NF-κB-miR-155 program, restores SHIP1/PU.1 differentiation, and creates a NOXA-dependent BCL-2 vulnerability	Pevonedistat plus azacitidine; azacitidine–venetoclax–pevonedistat triplets; biomarker-guided BCL-2 inhibition	Strongest clinical development among neddylation-targeted indications; early-phase activity was encouraging, although randomized studies have not consistently demonstrated survival benefit	(Swords et al., 2015; Swords et al., 2010; Khalife et al., 2015; Knorr et al., 2015; Cojocari et al., 2022)
Hematologic malignancies	DLBCL, mantle cell lymphoma, CLL, and other B-cell malignancies	Subtype-specific NF-κB dependence; CDT1-mediated replication licensing; BCL-xL, BIM, MCL-1, and stromal survival signaling	Produces subtype-specific NF-κB suppression in ABC-DLBCL or replication-licensing stress in GCB-DLBCL; disrupts stromal survival signaling in CLL.	Combination with rituximab, BTK inhibitors, or other B-cell receptor-directed therapies	Single-agent activity has been limited, whereas combination approaches have shown greater translational potential; response is likely to require molecular and lineage-based selection	(Milhollen et al., 2010; Czuczman et al., 2016; Wang et al., 2015; Godbersen et al., 2014; Shah et al., 2016)
Hematologic malignancies	Multiple myeloma	Canonical and noncanonical NF-κB signaling; proteostasis dependence	Suppresses canonical and noncanonical NF-κB signaling and exploits plasma-cell proteostasis dependence	Combination with bortezomib, lenalidomide–dexamethasone, daratumumab, or elotuzumab	Strong preclinical rationale, but clinical validation remains limited	(Muraoka et al., 2019; Shah et al., 2016)
Gastrointestinal cancers	Colorectal cancer	NAE–CRL signaling; DCAF13–CRL4; UBE2M–USP39–PABPC1–CCNB1 axis	Enhances TOP1-DNA-lesion toxicity and disrupts the UBE2M-USP39-PABPC1-CCNB1 translation axis	Combination with irinotecan or other TOP1 inhibitors; selective targeting of UBE2M-dependent signaling	Robust preclinical evidence in cell lines, patient-derived organoids, and xenografts; clinical validation is still required	(Mi et al., 2025; Sun et al., 2023; Wang Z. et al., 2026)
Gastrointestinal cancers	Gastric cancer	CUL1-dependent CRL activity; NAE1-mediated TFR1 neddylation	Defines an H. pylori-induced reduction of NAE1-TFR1 signaling that limits ferroptosis, highlighting a context-specific caveat for pathway inhibition	Pevonedistat-based chemotherapy combinations; exploration of NAE1–TFR1-directed ferroptosis strategies	Early clinical evaluation of pevonedistat plus CapeOX has demonstrated feasibility and preliminary activity; biomarker development remains necessary	(Shoji et al. 2025; Wang et al., 2025; Lan et al., 2016)
Gastrointestinal cancers	Pancreatic cancer	Global cullin neddylation; UBE2F–CRL5-ASB11–DIRAS2 axis	Restores DIRAS2 through blockade of the UBE2F-CRL5-ASB11 module and sensitizes tumors to gemcitabine or radiation	Combination with gemcitabine or radiotherapy; selective inhibition of the UBE2F–CRL5 module	Predominantly preclinical; selective E2–CRL targeting may offer a wider therapeutic window than global NAE inhibition	(Chang et al., 2024; Li et al., 2017; Wei et al., 2012)
Gastrointestinal cancers	Hepatocellular carcinoma	UBE2M-dependent β-catenin/cyclin D1 and p53/MDM2 signaling	Attenuates UBE2M-driven β-catenin/cyclin D1 and p53/MDM2 oncogenic signaling	Development of UBE2M-selective inhibitors or biomarker-guided pathway blockade	Mechanistically compelling but predominantly preclinical	(Zhang et al., 2020; Kim et al., 2021; Wu et al., 2025)
Gastrointestinal cancers	Intrahepatic cholangiocarcinoma	NAE1/UBA3–UBE2M–cullin hyperneddylation	Suppresses NAE1-dependent hyperneddylation and perturbs tumor-stroma crosstalk	Pevonedistat monotherapy or combination with cisplatin	Strong preclinical evidence supports pathway hyperactivation as a disease-associated vulnerability; clinical studies are needed	(Gao et al., 2014; Olaizola et al., 2022)
Genitourinary tumors	ccRCC/KIRC	CUL1–CRL signaling; neddylation-related immune and inflammatory networks; CUL4B–FBP1–HIF axis	Restores nuclear FBP1 by blocking CUL4B-dependent degradation, thereby constraining HIF-driven glycolysis and redox adaptation	Pevonedistat-based combinations with metabolic or redox-targeted agents; biomarker selection using neddylation-related molecular signatures	Extensive mechanistic evidence but limited disease-specific clinical evaluation; metabolic and immune biomarkers require functional and prospective validation	(Yang Y. et al., 2025; Tong et al., 2017; Liu et al., 2024b)
Genitourinary tumors	Bladder cancer	NAE–CRL signaling; DNA-damage and JNK–Bcl-xL apoptotic pathways	Enhances cisplatin response through DNA damage, JNK activation, and Bcl-xL suppression	Combination with cisplatin; development of neddylation-related prognostic or predictive signatures	Preclinical efficacy is promising, but proposed gene signatures require external and prospective validation	(Kuo et al., 2015; Ho et al., 2015; Xiaolong et al., 2025)
Genitourinary tumors	Prostate cancer	CRL-dependent regulation of AR/AR-V7 signaling; UBE2M/UBC12-dependent checkpoint control	Represses AR/AR-V7 transcription and invasion and strengthens the UBC12-WEE1 radiation-checkpoint vulnerability	Combination with androgen-receptor antagonists, androgen deprivation, or radiotherapy	Strong preclinical rationale for androgen-axis and radiation combinations; clinical evaluation remains limited	(Zhou X. et al., 2020; Wang et al., 2014; Wang et al., 2016)
Genitourinary tumors	Ovarian cancer	Neddylation-dependent DNA-damage tolerance and apoptotic regulation	Restores platinum sensitivity but can antagonize paclitaxel-induced microtubule polymerization, demonstrating partner-specific effects	Biomarker-guided platinum combinations; avoidance of empirically selected cytotoxic partners	Demonstrates marked treatment-context dependence, emphasizing the need for mechanism-based partner selection	(Jazaeri et al., 2013; Nawrocki et al., 2013; Hong et al., 2019)
Thoracic cancers	NSCLC and lung adenocarcinoma	Global NAE–CRL activity; UBE2M/UBC12; p21, p27, WEE1, and NOXA turnover	Blocks UBE2F-CUL5-mediated NOXA degradation and reverses platinum/radiation resistance; UBE2M depletion may bypass NAE-inhibitor resistance	Pevonedistat plus docetaxel; E2-directed inhibition in MLN4924-resistant tumors	Clinical activity has been observed with pevonedistat–docetaxel, whereas selective UBE2M targeting remains preclinical	(Li L. et al., 2019; Li et al., 2014; Zhou W. et al., 2017; Zhou L. et al., 2020; Qin A. et al., 2024)
Thoracic cancers	SCLC and extrapulmonary small-cell carcinoma	Neddylation-dependent maintenance of the neuroendocrine state	Disrupts neddylation-dependent maintenance of neuroendocrine lineage identity	NAE inhibition in molecularly defined small-cell tumors	Supported by genetically engineered and patient-derived models; clinical validation is needed	(Norton et al., 2021)
Thoracic and head-and-neck squamous cancers	HNSCC, LUSC, and DCUN1D1-amplified squamous tumors	NAE–CRL signaling; c-Myc–NOXA axis; TOP1 turnover; DCUN1D1/DCN1–UBE2M interaction	Engages c-Myc-NOXA signaling, stabilizes TOP1 for TOP1-inhibitor combinations, and exploits DCUN1D1-amplified squamous dependencies	Combination with radiotherapy or TOP1 inhibitors; selective DCN1–UBE2M inhibitors in DCUN1D1-amplified tumors	Strong preclinical evidence; DCUN1D1 amplification represents a promising biomarker for selective downstream inhibition	(Kim et al., 2019; Vanderdys et al., 2018; Zhang et al., 2019; Gu et al., 2023; Sarkaria et al., 2006; Yoo et al., 2012; Wang et al., 2013; Chatziandreou et al., 2021)
Brain tumors	Glioblastoma and glioblastoma stem-like cells	Hyperactivated NAE–CRL signaling; ERK/AKT-associated sensitivity; adaptive PD-L1 induction	Targets ERK/AKT-high glioblastoma stem-like cells and creates PD-L1, temozolomide, and oncolytic-virus combination opportunities	Combination with temozolomide, PD-1/PD-L1 blockade, or oncolytic virotherapy	Extensive preclinical evidence supports combination-based development; biomarker-guided clinical evaluation remains necessary	(Hua et al., 2015; Brandt et al., 2023; Guo, 2023)
Pediatric tumors	Neuroblastoma, medulloblastoma, retinoblastoma, and recurrent pediatric solid/CNS tumors	Replication-stress dependence; p53-modulated responses; KBTBD4–CRL3 adaptor biology; SKP2–CRL signaling	Exploits p53-modulated neuroblastoma sensitivity, KBTBD4-CoREST adaptor biology, and localized SKP2-CRL dependence in retinoblastoma	Pevonedistat plus irinotecan and temozolomide; molecularly selective CRL-adaptor targeting; intravitreal delivery in retinoblastoma	A pediatric phase I study established a recommended phase II dose for pevonedistat-based chemotherapy; efficacy remains preliminary and tumor-context dependent	(Foster et al., 2024; Foster et al., 2021; Smith et al., 2012; Chen et al., 2022; Aubry et al., 2020; Vriend and Rastegar, 2020)

AR, androgen receptor; BCL-2, B-cell lymphoma 2; BTK, bruton tyrosine kinase; ccRCC, clear cell renal cell carcinoma; CLL, chronic lymphocytic leukemia; CNS, central nervous system; CRC, colorectal cancer; DLBCL, diffuse large B-cell lymphoma; EMT, epithelial–mesenchymal transition; HCC, hepatocellular carcinoma; HIF, hypoxia-inducible factor; HNSCC, head-and-neck squamous cell carcinoma; KIRC, kidney renal clear cell carcinoma; LUSC, lung squamous cell carcinoma; MDS, myelodysplastic syndrome; NAE, NEDD8-activating enzyme; NSCLC, non-small-cell lung cancer; SCLC, small-cell lung cancer; TOP1, topoisomerase I.

## 6 Combination strategies

### 6.1 Chemotherapy combinations

Combining neddylation inhibitors with chemotherapy is mechanistically attractive because NAE inhibition disrupts CRL-dependent protein turnover and promotes the accumulation of replication-licensing and checkpoint regulators. These changes increase DNA re-replication stress, intensify checkpoint activation, and reduce the capacity of tumor cells to tolerate or repair chemotherapy-induced DNA damage (Garcia et al., 2014; Paiva et al., 2015). In a systematic study published in 2014, it was demonstrated that MLN4924 synergizes with mitomycin C through mechanisms involving ATR, BRCA1/BRCA2, and chromatin-regulatory pathways (Garcia et al., 2014). These findings indicated that neddylation inhibition compromises recovery from genotoxic stress rather than merely producing additive, nonspecific cytotoxicity (Garcia et al., 2014). Similarly, in primary chronic lymphocytic leukemia cells, MLN4924 induces CDT1 dysregulation, DNA damage, checkpoint activation, and G2-phase arrest, whereas combination with the alkylating agents bendamustine or chlorambucil further increases DNA damage and apoptosis (Paiva et al., 2015). Platinum-based combinations are especially well supported. Study was showed that MLN4924 resensitized cisplatin-resistant ovarian cancer models by increasing DNA damage, oxidative stress, and pro-apoptotic signaling, while another study was reported in 2023 that pevonedistat synergized with carboplatin in renal medullary carcinoma by inhibiting DNA-damage repair (Nawrocki et al., 2013; Shapiro et al., 2023). A related HNSCC study showed that pevonedistat or CUL4A inhibition enhanced cisplatin-induced DNA damage and apoptosis through downregulation of DDB2, a nucleotide-excision-repair factor involved in cisplatin resistance (Jones et al., 2022). These findings suggest that the efficacy of platinum combinations may depend on disabling CRL-regulated repair and stress-adaptation pathways. The clinical feasibility of combining pevonedistat with conventional cytotoxic regimens has also been explored. A phase Ib study in advanced solid tumors showed that pevonedistat could be administered in combination with docetaxel or carboplatin plus paclitaxel. However, the gemcitabine-containing arm was limited by tolerability, demonstrating that mechanistic synergy does not necessarily translate into an acceptable therapeutic window and that toxicity is highly dependent on the accompanying chemotherapy regimen (Lockhart et al., 2019).

Combinations with topoisomerase I (TOP1) inhibitors provide replication-stress amplification. Sun et al. reported in 2023 that pevonedistat synergizes with irinotecan or its active metabolite SN38 in colorectal cancer cells, patient-derived organoids, and xenograft models. Mechanistically, NAE inhibition impaired DCAF13–CRL4 activity and reduced the repair of TOP1–DNA protein crosslinks generated during replication, thereby converting TOP1-induced lesions into persistent cytotoxic damage (Sun et al., 2023). Temozolomide combinations are most relevant to glioblastoma and pediatric CNS/solid tumors; MLN4924 resensitized temozolomide-resistant glioblastoma cells, whereas the 2024 pediatric ADVL1615 phase I study found that pevonedistat plus irinotecan and temozolomide was tolerable in children with recurrent or refractory solid/CNS tumors and established a recommended phase II dose of 35 mg/m^2^ (Foster et al., 2024; Brandt et al., 2023). Hypomethylating-agent combinations are most developed in AML/MDS because azacitidine or decitabine can create epigenetic and replication-associated stress that converges with CRL-substrate accumulation after NAE inhibition (Visconte et al., 2016; Swords et al., 2018). Quantitative proteomics in AML cells were showed that Quantitative proteomics identified pharmacodynamic changes supporting the combination of MLN4924 with azacitidine in AML cells (Visconte et al., 2016), and a phase Ib study demonstrated the feasibility of pevonedistat plus azacitidine in older patients with treatment-naive AML (Swords et al., 2018). More recently, Cojocari et al. showed in 2022 that combined pevonedistat and azacitidine treatment induces greater NOXA accumulation than either agent alone and enhances venetoclax-mediated apoptosis in AML models (Cojocari et al., 2022). These findings indicate that the therapeutic effects of chemotherapy or epigenetic therapy may be further strengthened by incorporating agents that directly exploit treatment-induced apoptotic priming.

### 6.2 Radiotherapy combinations

Combining neddylation inhibitors with radiotherapy is supported by a strong mechanistic rationale because ionizing radiation induces DNA double-strand breaks (DSBs), whereas inhibition of NAE disrupts CRL-dependent regulation of replication licensing, cell-cycle checkpoints, and DNA damage responses. By stabilizing substrates such as CDT1, p21, p27, and WEE1, MLN4924 can convert radiation-induced DNA lesions into persistent replication stress, prolonged checkpoint activation, senescence, or apoptosis (Mladenov et al., 2013; Blank et al., 2013). NAE inhibition induces DNA re-replication and activates DNA damage checkpoints, thereby providing a mechanistic basis for combining neddylation blockade with radiation-induced genotoxic stress (Blank et al., 2013). Preclinical studies across multiple tumor types have consistently shown that neddylation inhibition can enhance radiation response through disruption of cell-cycle and DNA repair programs. In pancreatic cancer, MLN4924 inhibits cullin neddylation, increases and prolongs DNA damage signaling, enhances G2/M arrest, and improves the antitumor efficacy of irradiation in xenograft models (Wei et al., 2012). Similar effects have been observed in breast and colorectal cancer. Yang et al. reported that MLN4924 enhances breast cancer radiosensitivity through a p21-dependent mechanism, whereas Wan et al. showed that p27 accumulation contributes to increased radiation-induced G2/M arrest, apoptosis, and DNA damage responses in colorectal cancer cells (Yang et al., 2012; Wan et al., 2016). In HNSCC, pevonedistat increased DNA re-replication and replication-associated damage, thereby suppressing tumor growth and enhancing the response to irradiation (Vanderdys et al., 2018). Likewise, in hormone-resistant prostate cancer, either pharmacological inhibition with MLN4924 or genetic depletion of UBC12 increased radiosensitivity through accumulation of WEE1, p21, and p27, resulting in reinforced G2-phase arrest, greater DNA damage, and enhanced apoptosis (Wang et al., 2016).

More recent evidence indicates that radiosensitization may also be achieved through selective inhibition of downstream neddylation modules rather than global suppression of NAE activity. In lung cancer, researchers identified HA-9104 as a small-molecule inhibitor of the UBE2F–CRL5 axis. HA-9104 suppressed CUL5 neddylation, induced DNA damage and G2/M arrest, and enhanced radiosensitivity in both cellular and xenograft models, thereby providing proof of concept for a more pathway-restricted radiosensitizing strategy (Xu T. et al., 2022). In PAX3–FOXO1 fusion-positive rhabdomyosarcoma, a 2025 study further demonstrated that MLN4924 increases irradiation-induced DSBs and γH2AX accumulation while reducing RAD51 expression and DNA-PKcs activation. In cultured fusion-positive rhabdomyosarcoma cells, the combination enhanced apoptosis and inhibited clonogenic and spheroid growth. Reduced RAD51 and DNA-PKcs activation were consistent with impaired DNA-repair responses; MLN4924 monotherapy also inhibited tumor growth in vivo (Aiello et al., 2025). Collectively, these findings support a model in which neddylation inhibition enhances radiosensitivity through several convergent mechanisms, including induction of DNA re-replication, stabilization of cell-cycle inhibitors, reinforcement of G2/M arrest, disruption of checkpoint recovery, suppression of DNA repair capacity, and prolonged persistence of lethal DNA lesions (Xu T. et al., 2022; Wei et al., 2012; Blank et al., 2013; Aiello et al., 2025). The relative contribution of each mechanism is likely to vary according to tumor lineage, baseline DNA repair capacity, p53 status, and dependence on specific CRL modules. Despite encouraging preclinical activity, the therapeutic ratio remains a central translational concern. Normal tissues are also vulnerable to radiation-induced DNA damage, and the clinical value of a radiosensitizer depends on whether enhancement of tumor-cell killing exceeds sensitization of dose-limiting normal tissues, including the mucosa, skin, intestine, lung, and bone marrow (Kim et al., 2014; Larrey and Pathak, 2022). This consideration is particularly relevant to pevonedistat because systemic NAE inhibition affects a fundamental post-translational modification pathway with broad physiological functions. Phase I studies in advanced solid tumors have reported dose-limiting systemic toxicities and treatment-associated markers of organ injury at higher exposure levels, even in the absence of concurrent radiotherapy (Bhatia et al., 2016). Accordingly, radiotherapy combinations with neddylation inhibitors should currently be regarded as highly promising but predominantly preclinical strategies. We propose evaluating pharmacodynamic measures of cullin deneddylation, checkpoint disruption, and DNA repair inhibition to guide dose and schedule studies; their value for patient selection in radiotherapy combinations remains to be established.

### 6.3 Targeted therapy combinations

Combining neddylation inhibitors with molecularly targeted agents is mechanistically attractive because inhibition of NAE or downstream E2/co-E3 modules alters CRL-dependent substrate turnover, disrupts proteostasis, resets apoptotic thresholds, impairs DNA repair, and modifies oncogenic signaling networks. These effects may create or intensify therapeutic dependencies on the proteasome, BCL-2 family proteins, kinase signaling, PARP-mediated DNA repair, and the PI3K/AKT/mTOR pathway. In multiple myeloma, MLN4924 induces REDD1, suppresses AKT/mTOR signaling, and synergizes with bortezomib, including under conditions in which IL-6 and IGF-1 provide prosurvival support (Gu et al., 2014). This rationale was subsequently extended to TAS4464. TAS4464 inhibits both canonical and noncanonical NF-κB signaling and enhances the activity of bortezomib, lenalidomide plus dexamethasone, daratumumab, and elotuzumab (Muraoka et al., 2019). These findings suggest that NAE inhibition may cooperate not only with proteasome blockade but also with immunomodulatory and antibody-based therapies by simultaneously disrupting proteostasis and survival signaling. In prostate cancer, the natural-product-derived neddylation modulator flavokawain B was shown to promote SKP2 degradation and enhance the antitumor effects of bortezomib (Li X. et al., 2019). Nevertheless, such compounds remain exploratory preclinical tools rather than validated therapeutic agents. Combinations with BCL-2-family inhibitors are supported by a complementary apoptosis-priming mechanism. In AML, MLN4924 induces NOXA expression and synergizes with the BCL-2 inhibitors ABT-199/venetoclax and ABT-263/navitoclax, thereby reducing the apoptotic buffering capacity provided by anti-apoptotic BCL-2-family proteins (Knorr et al., 2015). This concept has advanced into clinical investigation. A 2024 phase I study evaluating pevonedistat in combination with azacitidine and venetoclax in relapsed or refractory AML reported that the regimen was feasible and demonstrated encouraging preliminary activity, supporting further evaluation of neddylation inhibition as an apoptosis-sensitizing component of venetoclax-based therapy (Murthy et al., 2024). These findings indicate that baseline dependence on BCL-2-family proteins and the capacity for NOXA induction may represent candidate biomarkers of response.

Kinase-inhibitor combinations are mechanistically heterogeneous and are therefore better considered according to the signaling pathway being targeted. In B-cell malignancies, pevonedistat may complement inhibition of B-cell receptor signaling. Pevonedistat combined with the Bruton’s tyrosine kinase inhibitor ibrutinib was tolerable and exhibited promising preliminary activity in relapsed or refractory non-Hodgkin lymphoma and chronic lymphocytic leukemia (Torka et al., 2023). The biological rationale is based on simultaneous disruption of NF-κB-associated survival signaling by NAE inhibition and attenuation of B-cell receptor signaling by BTK blockade. In Philadelphia chromosome-positive leukemia, MLN4924 enhances imatinib-induced DNA damage and replication stress and promotes apoptosis by shifting the balance between MCL1 and NOXA (Bahjat et al., 2019). These findings suggest that neddylation inhibition may deepen responses to oncogene-directed kinase therapy by increasing replication-associated stress and weakening anti-apoptotic adaptation. PARP-inhibitor combinations exploit the effects of neddylation inhibition on homologous recombination and DNA damage repair. Guo et al. demonstrated that MLN4924 impairs recruitment of BRCA1-complex components to sites of DNA damage and synergizes with olaparib in non-small cell lung cancer cells, resulting in defective DNA repair and enhanced suppression of tumor-cell growth (Guo et al., 2017). This interaction provides a potential synthetic-lethal framework in which neddylation blockade creates a state of impaired DNA repair that increases dependence on PARP-mediated compensatory pathways.

PI3K/AKT/mTOR-pathway combinations are less clinically mature but biologically plausible. It was reported that the AKT inhibitor MK-2206 sensitized breast cancer cells to MLN4924 by enhancing DNA damage and apoptosis, while Jiang et al. showed in 2024 that MLN4924 disrupts NEDD8/CUL4-dependent regulation of TSC2 and inactivates mTOR signaling in HNSCC, providing a mechanistic basis for exploring rational cotargeting of neddylation and PI3K/AKT/mTOR signaling (Jiang et al., 2024; Chen et al., 2018). A related immuno-kinase study showed that MLN4924-induced PD-L1 upregulation is mediated by the MEK–JNK–AP1 axis and that combining MLN4924 with a MEK inhibitor enhanced tumor suppression, reinforcing that kinase combinations may be needed not only for tumor-cell signaling blockade but also to counteract adaptive immune-evasive responses (Zhang et al., 2022). Overall, targeted combinations with neddylation inhibitors are most advanced for proteasome inhibitors, BCL-2 inhibitors, and selected kinase inhibitors, whereas PARP and PI3K/AKT/mTOR combinations remain mechanistically compelling but require stronger *in vivo* validation, biomarker selection, and toxicity assessment before clinical prioritization.

### 6.4 Immunotherapy combinations

Combining neddylation inhibitors with immunotherapy is biologically compelling because the NEDD8–CRL axis regulates not only tumor-cell survival but also inflammatory signaling, cytokine and chemokine production, antigen presentation, and immune-cell function within the tumor microenvironment (Liu et al., 2024a; Zhou L. et al., 2019). However, the immunological consequences of pathway inhibition are highly context-dependent and may vary according to tumor lineage, immune-cell subset, drug exposure, and treatment schedule. Accordingly, neddylation blockade should not be viewed as intrinsically immune-stimulatory but rather as an intervention capable of reshaping multiple components of antitumor immunity. Several studies have demonstrated that NAE inhibition can enhance T-cell-mediated antitumor responses. In T cells derived from patients with chronic lymphocytic leukemia, it was reported that pevonedistat reduced regulatory T-cell differentiation, promoted a Th1-like phenotype, and increased IFN-γ production, suggesting that neddylation inhibition may partially reverse tumor-associated immune dysfunction (Best et al., 2021). More direct evidence showed that pharmacological NAE inhibition reinvigorates CD8^+^ T-cell function in lymphoid neoplasia. Treatment increased IFN-γ and TNF-α production, enhanced cytotoxic activity, induced CD8^+^ T-cell-dependent tumor suppression in syngeneic lymphoma models, and cooperated with PD-1 blockade (Wang et al., 2023). A genome-wide CRISPR co-culture study published in 2024 further identified NEDD8 loss as a tumor-cell vulnerability to immune-checkpoint therapy in triple-negative breast cancer. NEDD8-deficient tumor cells exhibited increased immunogenicity and HLA-DR expression, whereas Nedd8-deficient EO771 tumors underwent CD8^+^ T-cell-dependent regression following PD-1 blockade in immunocompetent mice (Papakyriacou et al., 2024). Tumor proteostasis may represent an additional mechanistic link between neddylation inhibition and immune-checkpoint sensitivity. In mismatch repair-deficient or microsatellite instability-high tumors, malignant cells depend on the neddylation pathway to eliminate destabilized mutant proteins. MLN4924 increased the accumulation of misfolded protein aggregates and promoted immunogenic cell death, thereby providing a rationale for combining NAE inhibition with PD-1 blockade in tumors characterized by proteome instability (McGrail et al., 2020). Neddylation inhibition may also reduce myeloid-mediated immunosuppression. Zhou et al. demonstrated in 2022 that pathway activation promotes myeloid-derived suppressor cell (MDSC) recruitment in lung cancer through NF-κB-dependent murine CXCL5 (mCXCL5; the prefix “m” denotes mouse; human homolog: CXCL6) signaling (Zhou L. et al., 2022). Pharmacological inhibition reduced MDSC infiltration and suppressed Lewis lung carcinoma growth, suggesting that neddylation blockade may remodel an immunosuppressive myeloid niche and improve responsiveness to immunotherapy (Zhou L. et al., 2022).

Despite these immune-enhancing effects, neddylation inhibition can also activate compensatory immune-evasion pathways. In glioblastoma, MLN4924 increases PD-L1 expression and that addition of PD-L1 blockade enhances the antitumor activity of pevonedistat (Zhou S. et al., 2019). Although this finding supports combination therapy, it also indicates that NAE inhibition can induce an adaptive checkpoint response that limits T-cell-mediated tumor killing. Study have shown that MLN4924 increases PD-L1 expression across multiple cancer-cell types through the MEK–JNK–AP1 signaling axis. This response reduced T-cell cytotoxicity, whereas anti-PD-L1 treatment or MEK inhibition restored antitumor activity (Zhang et al., 2022). These findings suggest that treatment-induced PD-L1 upregulation may serve both as a resistance mechanism and as a biomarker for rational immune-checkpoint combinations. The effects of neddylation inhibition on antigen presentation are similarly complex. It was reported in 2022 that MLN4924 impairs MHC class I presentation of antigens derived from defective ribosomal products through a mechanism independent of direct neddylation of the antigenic proteins (Vijayasimha et al., 2022). This finding suggests that intact NEDD8 signaling may support selected pathways of direct antigen presentation. Professional antigen-presenting cells may also be adversely affected. Cheng et al. showed that MLN4924 suppresses dendritic-cell maturation, reduces production of TNF-α, IL-6, and IL-12p70, decreases CD80 and CD86 expression, weakens allogeneic T-cell stimulation, and promotes DEPTOR accumulation with subsequent mTOR inhibition (Cheng et al., 2016). Thus, systemic pathway inhibition may impair dendritic-cell activation even when tumor-cell immunogenicity is increased. Direct effects on lymphocyte homeostasis also require careful consideration. Wu et al. demonstrated in 2022 that the UBE2M–RBX1–CRL neddylation module is essential for maintaining regulatory T-cell fitness and suppressive function (Wu et al., 2022). Although disruption of this pathway could reduce tumor-associated immune suppression, excessive impairment of regulatory T cells may also disturb immune homeostasis. More broadly, these findings indicate that neddylation inhibitors can alter antitumor immunity through simultaneous effects on tumor cells, cytotoxic lymphocytes, regulatory T cells, dendritic cells, and myeloid populations.

Based on these mechanism-driven combination therapy strategies, various pevonedistat-based regimens have advanced from preclinical evaluation to early- and late-stage clinical studies targeting hematologic malignancies and specific solid tumors. Table 2 summarizes the relevant clinical trials mentioned above, covering treatment regimens, study phases, trial identifiers, key efficacy and safety outcomes, and relevant references.

**TABLE 2:** Selected clinical trials of neddylation inhibitors in combination regimens and reported outcomes (updated through July 2026).

Combination category	Regimen and patient population	Phase and sample size	Trial identifier	Main clinical findings	Representative references
Chemotherapy	Pevonedistat plus docetaxel, carboplatin–paclitaxel, or gemcitabine in advanced solid tumors	Phase Ib; 54 response-evaluable patients	NCT01862328	The maximum tolerated dose of pevonedistat was 25 mg/m^2^ with docetaxel and 20 mg/m^2^ with carboplatin–paclitaxel. Objective response rates were 16% and 35%, respectively. The gemcitabine-containing arm was discontinued because of poor tolerability, including severe myelosuppression and one treatment-related death	(Lockhart et al., 2019)
Hypomethylating therapy	Pevonedistat plus azacitidine in older patients with treatment-naïve AML who were considered unsuitable for intensive induction therapy	Phase Ib; 64 patients	NCT01814826	The combination was clinically feasible and produced an overall response rate of 50%, including complete remission in a substantial proportion of patients. These findings established 20 mg/m^2^ pevonedistat on days 1, 3, and 5 as the recommended combination dose and supported subsequent randomized development	(Swords et al., 2018)
Hypomethylating therapy	Pevonedistat plus azacitidine *versus* azacitidine alone in higher-risk MDS, CMML, or low-blast AML	Randomized phase II; 120 patients	NCT02610777	Median event-free survival was 21.0 *versus* 16.6 months (HR, 0.665; *P* = 0.076), and median overall survival was 21.8 *versus* 19.0 months (HR, 0.802; *P* = 0.334). The combination generated a favorable efficacy signal, particularly in higher-risk MDS, without a substantial increase in toxicity, but the primary analysis did not reach conventional statistical significance	(Sekeres et al., 2021)
Hypomethylating therapy	Pevonedistat plus azacitidine *versus* azacitidine alone in newly diagnosed higher-risk MDS, CMML, or low-blast AML (PANTHER study)	Randomized phase III; 454 patients	NCT03268954	Median event-free survival was 17.7 months with the combination and 15.7 months with azacitidine alone. The addition of pevonedistat did not significantly improve event-free or overall survival, although the overall safety profile remained generally comparable between treatment groups	(Adès et al., 2022)
Hypomethylating/BCL-2-targeted therapy	Pevonedistat plus azacitidine and venetoclax in newly diagnosed secondary AML or MDS/CMML after hypomethylating-agent failure	Phase I/II	NCT03862157	The triplet showed encouraging activity in a very-high-risk population. In the MDS/CMML cohort, the overall response rate was 75%, including complete remission, marrow complete remission, and hematologic improvement. The most frequent grade 3–4 toxicities were infection, febrile neutropenia, and hypophosphatemia	(Short et al., 2023)
BCL-2-targeted therapy	Pevonedistat plus azacitidine and venetoclax in relapsed or refractory AML	Phase I; 16 patients	NCT04172844	The recommended phase II dose of pevonedistat was 20 mg/m^2^. The overall response rate was 46.7%; among patients without previous hypomethylating-agent/venetoclax exposure, 5 of 7 achieved complete remission. Frequent grade ≥3 toxicities included neutropenia, febrile neutropenia, anemia, hypertension, and sepsis	(Murthy et al., 2024)
BCL-2-targeted therapy	Azacitidine and venetoclax with or without pevonedistat in newly diagnosed AML unsuitable for intensive chemotherapy	Randomized phase II; 164 patients	NCT04266795	Complete remission rates were 45% with the pevonedistat triplet and 49% with azacitidine–venetoclax; composite complete remission rates were 77% and 72%, respectively. Pevonedistat did not improve event-free survival (HR, 0.99) or overall survival (HR, 1.42), and the study was terminated early following the negative phase III PANTHER findings	(Short et al., 2025)
Chemotherapy	Pevonedistat plus irinotecan and temozolomide in pediatric patients with recurrent or refractory solid tumors or CNS tumors (ADVL1615)	Phase I	NCT03323034	The regimen was generally tolerable, and the recommended phase II dose of pevonedistat was established at 35 mg/m^2^ on days 1, 3, and 5 in combination with irinotecan and temozolomide. As a dose-escalation study, it was not designed to establish comparative efficacy	(Foster et al., 2024)
Chemotherapy	Pevonedistat plus docetaxel in previously treated advanced non-small-cell lung cancer	Phase II; 31 patients	NCT03228186	The objective response rate was 22%, median progression-free survival was 4.1 months, and median overall survival was 13.2 months. The regimen demonstrated manageable safety and evidence of antitumor activity in relapsed disease	(Qin A. et al., 2024)
Targeted therapy	Pevonedistat plus the BTK inhibitor ibrutinib in relapsed or refractory B-cell non-Hodgkin lymphoma or chronic lymphocytic leukemia	Phase I; 18 patients	NCT03479268	The estimated maximum tolerated dose of pevonedistat was 50 mg/m^2^. The overall response rate was 65%, including a 100% response rate among patients with mantle-cell lymphoma. The combination was generally tolerable, with bruising, diarrhea, and atrial fibrillation among the principal adverse events	(Torka et al., 2023)

BTK, bruton tyrosine kinase; CMML, chronic myelomonocytic leukemia; CNS, central nervous system; CR, complete remission; HR, hazard ratio; MDS, myelodysplastic syndrome; NAE, NEDD8-activating enzyme; ORR, objective/overall response rate, according to study definition; PD-1, programmed cell death protein 1; RP2D, recommended phase II, dose.

## 7 Challenges and limitations

### 7.1 Therapeutic window and systemic toxicity

A central limitation of neddylation-targeted therapy is the narrow and context-dependent therapeutic window created by the fact that NEDD8 modification regulates core cellular processes, including CRL activity, cell-cycle progression, signal transduction, stress adaptation, metabolism, and immune homeostasis (Zhang S. et al., 2024). Therefore, global inhibition of NAE can affect not only tumor cells but also normal proliferating tissues, hematopoietic compartments, immune cells, liver function, and adaptive stress-response pathways (Zhang S. et al., 2024; Tang et al., 2024). This concern is evident in clinical experience with pevonedistat. Phase I solid-tumor studies established pharmacodynamic pathway inhibition but also reported dose-limiting toxicities and adverse events requiring dose interruption or schedule optimization (Sarantopoulos et al., 2016). In combination regimens, toxicity becomes more clinically relevant; a phase Ib study of pevonedistat with docetaxel, carboplatin/paclitaxel, or gemcitabine reported cycle-1 dose-limiting toxicities including grade ≥3 liver enzyme elevations, febrile neutropenia, and thrombocytopenia, and the gemcitabine arm was discontinued because of poor tolerability (Lockhart et al., 2019). Expanded safety analyses in AML also highlighted hepatotoxicity and sepsis/multiorgan-failure syndromes as important serious toxicities requiring careful dose and schedule control (Swords et al., 2017). TAS4464 illustrates an even sharper translational barrier: although it is a potent NAE inhibitor with strong preclinical activity, its first-in-human phase I trial in advanced/metastatic solid tumors was limited by liver-function abnormalities, treatment discontinuations, and dose-limiting toxicities related to abnormal liver tests (Yamamoto et al., 2021). Combination strategies may increase efficacy but can also amplify hematologic and infectious risks, as a 2024 phase I study of pevonedistat plus azacitidine and venetoclax in relapsed/refractory AML reported grade ≥3 neutropenia, febrile neutropenia, anemia, thrombocytopenia, hypertension, and sepsis (Murthy et al., 2024). Immune toxicity and immune remodeling also require caution because neddylation is necessary for immune-cell fitness; recent work showed that neddylation maintains DN T-cell homeostasis in lupus models, while pevonedistat can reshape CLL-derived T-cell differentiation and cytokine production, indicating that systemic inhibition may have both therapeutically useful and potentially disruptive immune effects (Best et al., 2021; Zhang Y. et al., 2024).

### 7.2 Lack of validated predictive biomarkers

A second major limitation is the lack of validated predictive biomarkers capable of identifying patients most likely to benefit from neddylation inhibition. Early clinical enthusiasm was supported by phase II activity of pevonedistat plus azacitidine in higher-risk MDS/CMML or low-blast AML, but the phase III PANTHER trial did not improve event-free survival in the intent-to-treat population, underscoring that histology-based enrollment and pharmacodynamic target inhibition alone are insufficient for robust patient selection (Sekeres et al., 2021; Adès et al., 2022). Current development has therefore not yet established a biomarker-defined population analogous to EGFR-mutant lung cancer, BRCA-mutant PARP-inhibitor sensitivity, or BCR–ABL-driven TKI response. Several candidate biomarkers have emerged, but most remain preclinical or exploratory rather than clinically qualified: For instance, in glioblastoma, patient-derived glioblastoma stem-like cells with ERK/AKT pathway activation were more sensitive to MLN4924, suggesting that pathway activation status may help predict response in selected brain tumors (Han et al., 2019). Thus, future trials should integrate baseline neddylation activity, cullin-neddylation pharmacodynamics, CRL-substrate accumulation, genomic alterations, proteomic signatures, immune-state markers, and resistance mechanisms into prospective biomarker-stratified designs.

### 7.3 Adaptive resistance

Adaptive resistance is a major limitation of neddylation-targeted therapy because tumors may escape either by restoring NEDD8-pathway flux, rewiring downstream CRL-dependent signaling, or activating parallel survival programs. The most direct on-target resistance mechanism is mutation of the NAE catalytic subunit UBA3. Xu et al. showed in 2014 that MLN4924-resistant leukemia cells acquired UBA3 mutations such as I310N and Y352H, which reduced drug potency while preserving sufficient NAE function for leukemic-cell survival (Xu et al., 2014). Resistance may also emerge through adaptive metabolic rewiring rather than target mutation; for example, it was reported in 2022 that neddylation inhibition increased ASCT2/SLC1A5-dependent glutamine uptake through CRL3-SPOP inactivation, and another study was further reported in 2026 that AML cells can compensate for neddylation inhibition by upregulating glutamine metabolism, creating a rationale for cotargeting SLC1A5 (Zhou Q. et al., 2022; Wang Y. et al., 2026). Autophagy represents another adaptive stress response. MLN4924-triggered CRL inactivation induces protective autophagy in cancer cells, indicating that autophagy may buffer proteotoxic and replication stress and thereby reduce cytotoxic efficacy unless therapeutically cotargeted (Luo et al., 2012b). Alternative E2/E3-module usage may also shape resistance or lineage-specific escape; in lung cancer, platinum treatment induces UBE2F, which activates CUL5-related signaling and protects cells from platinum-induced apoptosis, while DCAF13–CRL4 has been implicated in repair of TOP1 DNA-protein crosslinks during irinotecan/SN38 exposure (Zhou L. et al., 2020; Sun et al., 2023). Anti-apoptotic pathway activation is particularly important because neddylation inhibition often relies on pro-apoptotic substrate accumulation; Knorr et al. showed that MLN4924-induced NOXA upregulation sensitizes AML cells to BCL-2/BCL-xL blockade, implying that persistent BCL-2-family buffering may blunt NAE-inhibitor cytotoxicity (Knorr et al., 2015).

### 7.4 Context-dependent biology

Context-dependent biology is equally important because neddylation inhibition does not simply accumulate “beneficial” tumor-suppressive substrates; it broadly disrupts CRL-mediated protein turnover and may stabilize proteins with either tumor-suppressive or tumor-promoting functions depending on genotype, lineage, immune context, and treatment schedule. In lymphoma, MLN4924 induces G2 arrest followed by either apoptosis or senescence in a cell-line-dependent manner, illustrating that the same pharmacologic perturbation can generate different terminal outcomes within a single tumor category (Wang et al., 2015). In ovarian cancer, MLN4924 attenuated paclitaxel-induced apoptosis and microtubule polymerization because UBE2M and UBE2F were required for paclitaxel-triggered tubulin polymerization, providing a clear example in which neddylation inhibition can antagonize rather than enhance a chemotherapy backbone (Hong et al., 2019). Broader mechanistic studies have also shown that MLN4924 can exert neddylation-independent effects, including EGFR dimerization, activation of RAS/MAPK and PI3K/AKT signaling, altered glycolysis through PKM2 tetramerization, and modulation of cilia biology, which complicates interpretation of phenotypes observed after drug exposure (Mao and Sun, 2020). This “double-edged” character was further emphasized by Tang et al., in 2024, who summarized how MLN4924 can stabilize both antitumor and protumor substrates, making outcome dependent on which CRL substrates dominate in a given biological context (Tang et al., 2024). At low concentrations or in specific cellular states, neddylation blockade may even promote stem-like features, as Zhou et al. reported that partial neddylation inhibition stimulated tumor-sphere formation, self-renewal, and stem-cell-like properties in breast cancer models (Zhou et al., 2016). Therefore, adaptive resistance and context-dependent biology should be treated as central barriers to clinical translation. Future studies need longitudinal resistance profiling, paired pre-/post-treatment biopsies, CRL-substrate proteomics, functional apoptosis profiling, autophagy and metabolic-flux assays, immune-competent models, and schedule-optimized combinations to determine whether neddylation inhibition produces lethal stress or compensatory survival in each tumor setting.

Collectively, these findings demonstrate that neddylation-pathway inhibition produces context-dependent outcomes determined by CRL-substrate accumulation, tumor-specific signaling dependencies, adaptive resistance mechanisms, immune remodeling, and systemic toxicity. As summarized in Figure 2, integrating these molecular consequences with mechanism-based combinations, predictive biomarkers, pharmacodynamic monitoring, and tumor-targeted delivery may convert broad pathway inhibition into a more precise effective therapeutic strategy.

**FIGURE 2:**
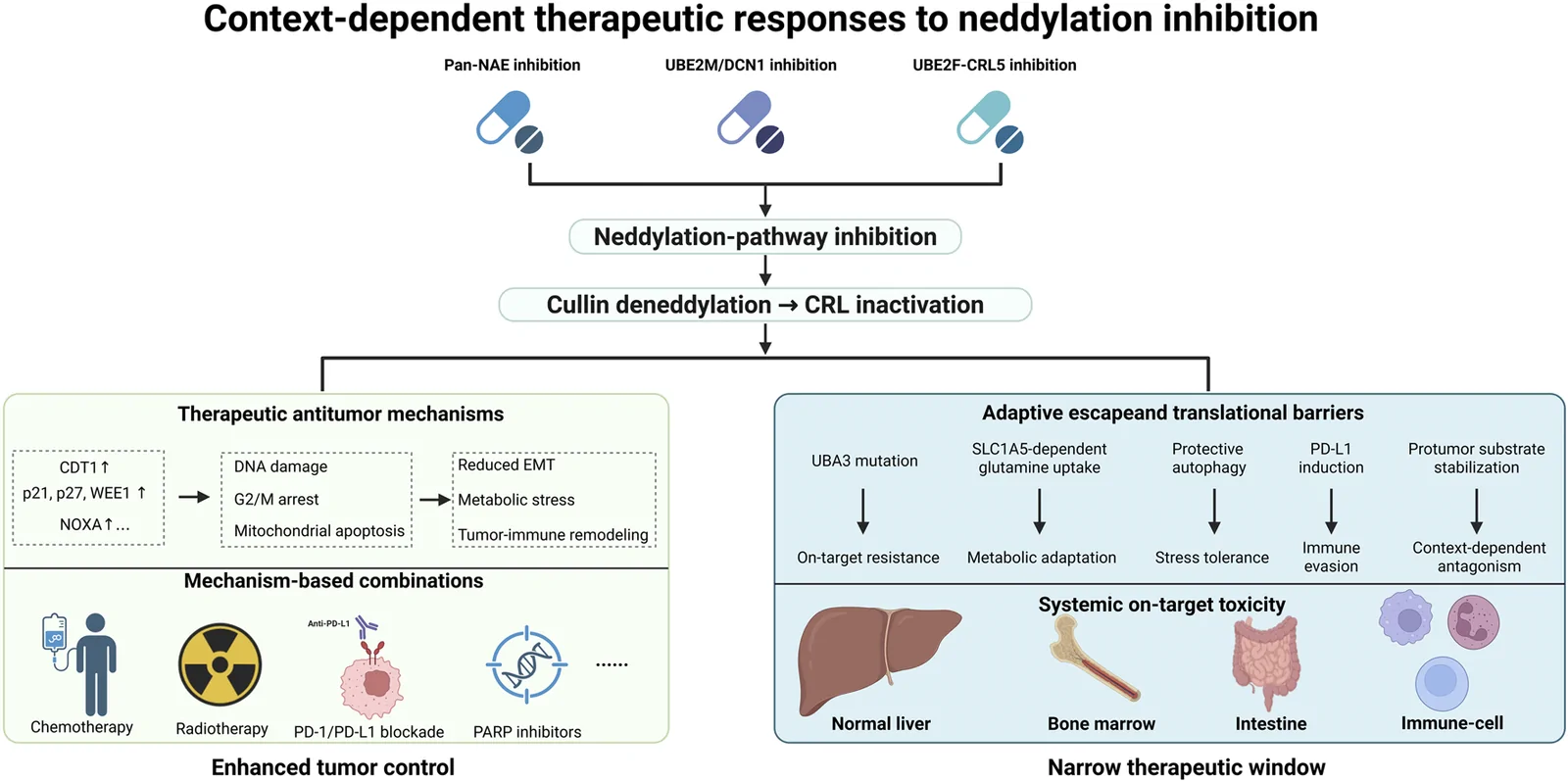
Context-dependent therapeutic responses to neddylation-pathway inhibition. Pharmacological inhibition of NAE or selective disruption of UBE2M/DCN1- and UBE2F–CRL5-dependent modules induces cullin deneddylation and CRL inactivation, resulting in context-dependent therapeutic and adaptive responses. Accumulation of CDT1 promotes DNA re-replication, replication stress, and DNA damage; stabilization of p21, p27, and WEE1 reinforces checkpoint activation, G2/M arrest, and senescence; NOXA induction lowers the mitochondrial apoptotic threshold; and phosphorylated IκBα accumulation suppresses NF-κB signaling. These events can additionally inhibit epithelial–mesenchymal transition and invasion, disrupt metabolic adaptation, and remodel tumor–immune interactions. Mechanistically compatible combinations with chemotherapy, radiotherapy, PARP inhibitors, BCL-2 inhibitors, proteasome or autophagy-targeted agents, and PD-1/PD-L1 blockade may enhance tumor control. Conversely, UBA3 mutations, SLC1A5-dependent glutamine uptake, protective autophagy, adaptive PD-L1 induction, and stabilization of protumor substrates can produce resistance or therapeutic antagonism. Systemic pathway inhibition may also damage the liver, bone marrow, intestine, and immune compartments, resulting in a narrow therapeutic window. Successful translation therefore requires biomarker-guided patient selection, CRL-substrate pharmacodynamic monitoring, immune-state assessment, schedule optimization, and tumor-targeted delivery. BCL-2, B-cell lymphoma 2; CRL, cullin–RING ligase; CDT1, chromatin licensing and DNA replication factor 1; EMT, epithelial–mesenchymal transition; NAE, NEDD8-activating enzyme; NOXA, phorbol-12-myristate-13-acetate-induced protein 1; PARP, poly(ADP-ribose) polymerase; PD-1, programmed cell death protein 1; PD-L1, programmed death-ligand 1; SLC1A5, solute carrier family 1 member 5; UBA3, ubiquitin-like modifier-activating enzyme 3; UBE2, ubiquitin-conjugating enzyme E2; WEE1, WEE1 G2 checkpoint kinase. Created in BioRender. Liu, D. (2026) https://BioRender.com/nomohl7.

## 8 Future perspectives

The next phase of neddylation-targeted cancer therapy should move beyond broad inhibition of NAE toward selective modulation of defined enzymatic and CRL modules. Although pevonedistat established neddylation as a pharmacologically tractable cancer vulnerability, pan-NAE inhibition affects numerous cullin and non-cullin substrates and consequently imposes substantial constraints on selectivity and therapeutic window. Future drug-development efforts may therefore benefit from targeting more restricted pathway nodes, including DCN1/DCUN1-family co-E3 ligases, UBE2M/UBC12-dependent CUL1–4 signaling, and UBE2F-dependent CUL5 activity (Qin X. et al., 2024; He et al., 2024). Emerging chemical probes support the feasibility of this strategy. DCN1 has been recognized as a structurally tractable co-E3 target, HA-9104 selectively inhibits the UBE2F–CRL5 axis and exhibits antitumor and radiosensitizing activity, and micafungin has been identified as a UBE2M-interacting compound capable of suppressing neddylation-dependent cancer-cell growth and migration (Xu T. et al., 2022; Mamun MAA. et al., 2023; He et al., 2024). However, future inhibitors should be optimized not only for biochemical potency but also for cullin preference, tumor-selective pathway dependence, pharmacokinetic properties, and reduced normal-tissue toxicity. Parallel advances in biomarker development will be required to identify tumors that are functionally dependent on specific CRL programs. Such “CRL-addicted” tumors may be characterized by elevated neddylation flux, dependence on particular substrate receptors, reliance on rapid turnover of tumor-suppressive proteins, or immune and metabolic states that create selective sensitivity to pathway inhibition. Multi-omics profiling, functional genetic screening, quantitative proteomics, and *ex vivo* drug-response testing should therefore be integrated to distinguish true pathway dependence from merely elevated expression of neddylation components. Early evidence supporting this approach includes a pevonedistat-response signature that predicted tumor-growth stabilization in patient-derived xenograft models and machine-learning-derived neddylation-associated signatures linked to prognosis, immune infiltration, and predicted treatment response in colorectal cancer (Picco et al., 2016; Yang G. et al., 2025). Future clinical trials should consequently move beyond histology-based enrollment and incorporate baseline NAE/E2/E3 expression, cullin-neddylation status, CRL-substrate profiles, genomic alterations, transcriptomic and proteomic signatures, and dynamic pharmacodynamic markers. Candidate on-treatment readouts include CDT1 and NRF2 accumulation, changes in cullin–NEDD8 conjugation, and formation of the pevonedistat–NEDD8 adduct. Additionally, combination therapy will likely remain the most practical route to clinical benefit, but partner selection should be driven by defined biological interactions rather than empirical screening alone. Ultimately, successful translation will require coordinated development of selective pathway inhibitors, biomarker-defined patient populations, mechanism-based combinations, tumor-directed delivery systems, and real-time pharmacodynamic monitoring. Such an integrated strategy may allow the field to progress from broad induction of pathway-wide cellular stress toward precise modulation of tumor-specific CRL dependencies.

## 9 Conclusion

Neddylation is a biologically compelling but clinically challenging therapeutic target because it coordinates CRL activity, non-cullin protein regulation, DNA replication licensing, DNA damage responses, apoptosis, inflammatory signaling, immune remodeling, and metabolic stress adaptation (Zhang S. et al., 2024; Fu and Wang, 2023). First-generation NAE inhibitors, particularly MLN4924, established the pharmacological tractability of this pathway by producing measurable suppression of neddylation and accumulation of downstream substrates in early clinical studies (Sarantopoulos et al., 2016; Shah et al., 2016). However, broad pathway inhibition has not yet translated into consistent clinical benefit. The phase III PANTHER trial provided an important cautionary example, as pevonedistat plus azacitidine failed to improve event-free survival over azacitidine alone in the intention-to-treat population with higher-risk MDS, chronic myelomonocytic leukemia, or low-blast AML despite encouraging earlier-phase findings (Adès et al., 2022). These outcomes indicate that the principal barriers are not insufficient biological rationale but inadequate patient selection, extensive pathway complexity, context-dependent CRL-substrate regulation, adaptive resistance, bidirectional immune effects, and therapeutic-window limitations (Zhang S. et al., 2024; Fu and Wang, 2023).

Future development should therefore move beyond empiric pan-NAE inhibition toward biomarker-guided and tumor-context-specific strategies. Identification of functionally “CRL-addicted” tumors will require integration of multi-omics profiling, cullin-neddylation status, quantitative CRL-substrate analysis, dynamic pharmacodynamic markers, resistance mechanisms, and immune-state assessment (Qin X. et al., 2024). More selective inhibition of defined pathway branches—including DCN1/DCUN1 co-E3 ligases, UBE2M-dependent CUL1–4 signaling, UBE2F-dependent CUL5 activity, and potentially individual cullin modules—may improve therapeutic precision and widen the therapeutic window relative to global NAE blockade (Mamun M. et al., 2023). Such an approach may enable neddylation-targeted therapy to evolve from broad induction of cellular stress into precision modulation of tumor-specific CRL dependencies.

## Glossary

AML: Acute myeloid leukemia
BCL-xL: B-cell lymphoma-extra large
ccRCC: Clear cell renal cell carcinoma
CLL: Chronic lymphocytic leukemia
CMML: Chronic myelomonocytic leukemia
CNS: Central nervous system
COP9: Constitutive photomorphogenesis 9
CRL: Cullin–RING ligase
CSN: COP9 signalosome
DCAF: DDB1- and CUL4-associated factor
DCN1: Defective in cullin neddylation 1
DLBCL: Diffuse large B-cell lymphoma
DSB: DNA double-strand break
E1: NEDD8-activating enzyme
E2: NEDD8-conjugating enzyme
E2F1: E2F transcription factor 1
E3: NEDD8 ligase
EGFR: Epidermal growth factor receptor
EMT: Epithelial–mesenchymal transition
ERα: Estrogen receptor alpha
ERK: Extracellular signal-regulated kinase
FBP1: Fructose-1,6-bisphosphatase 1
HCC: Hepatocellular carcinoma
HIF: Hypoxia-inducible factor
HNSCC: Head-and-neck squamous cell carcinoma
KIRC: Kidney renal clear cell carcinoma
LUSC: Lung squamous cell carcinoma
MDS: Myelodysplastic syndrome
MDSC: Myeloid-derived suppressor cell
mCXCL5: Murine C-X-C motif chemokine ligand 5 (human homolog: CXCL6)
MEK: Mitogen-activated protein kinase kinase
MHC: Major histocompatibility complex
NAE: NEDD8-activating enzyme
NEDD8: Neural precursor cell expressed, developmentally downregulated 8
NSCLC: Non-small-cell lung cancer
PABPC1: Poly(A)-binding protein cytoplasmic 1
SCLC: Small-cell lung cancer
SENP8: Sentrin-specific protease 8
VEGF: Vascular endothelial growth factor
VHL: Von Hippel–Lindau
